# Reversal of tyrosine-linked ADP-ribosylation by ARH3 and PARG

**DOI:** 10.1016/j.jbc.2024.107838

**Published:** 2024-09-27

**Authors:** Johannes Gregor Matthias Rack, Jim Voorneveld, Edoardo José Longarini, Sven Wijngaarden, Kang Zhu, Alessandra Peters, Jia Jhing Sia, Evgeniia Prokhorova, Dragana Ahel, Ivan Matić, Dmitri V. Filippov, Ivan Ahel

**Affiliations:** 1 MRC Centre for Medical Mycology, University of Exeter, Exeter, UK; 2 Leiden Institute of Chemistry, Leiden University, Leiden, The Netherlands; 3 Research Group of Proteomics and ADP-ribosylation Signalling, Max Planck Institute for Biology of Ageing, Cologne, Germany; 4 Sir William Dunn School of Pathology, University of Oxford, Oxford, UK; 5 Cologne Excellence Cluster for Stress Responses in Ageing-Associated Diseases (CECAD), University of Cologne, Cologne, Germany

**Keywords:** mass spectrometry, peptide synthesis, (ADP-ribosyl)hydrolase, PARP, DNA damage, HPF1

## Abstract

ADP-ribosylation is an ancient posttranslational modification with exceptional versatility in terms of breadth of modification targets including at least seven different amino acid side chains, various moieties on nucleic acids, and a variety of small chemical compounds. The spatiotemporal signaling dynamic of the different modification variations is tightly regulated and depends on the writers, erases, and readers of each type. Among these, tyrosine ADP-ribosylation (Tyr-ADPr) has been consistently detected as a novel modification type, but systematic analysis of its potential physiological role, modification establishment, and reversal are still lacking. Here we present a re-analysis of recent ADP-ribosylome data and show that Tyr-ADPr sites are conserved and enriched among ribosome biogenesis and mRNA processing proteins and that these sites are affected by the status of the (ADP-ribosyl)hydrolase ARH3. To facilitate the study of Tyr-ADPr, we establish methodologies for the synthesis of well-defined Tyr-ADPr peptides and with these could show that Tyr-ADPr is reversed both by ARH3 and PARG enzymes. Together, our work lays the foundation for the future exploration of the Tyr-ADPr.

ADP-ribosylation (ADPr) is an ancient modification found in all domains of life, which primarily targets proteins and nucleic acids (1, 2, 3). ADPr dynamics is tightly regulated by several means including signal-dependent (ADP-ribosyl)transferase (ART) activation, the physiochemical nature of the acceptor site, such as glutamates, aspartates, and serines, and degradation *via* a variety of hydrolases (1, 2, 3, 4, 5). This ability to fine-tune the ADPr signaling response allows for the regulation of a plethora of other cellular processes including DNA damage response, gene expression, intracellular signaling, replication, ribosome biogenesis, and RNA processing (2, 3, 6, 7). During times of genotoxic stress, the majority of the ADPr signal is created by the ARTs PARP1 and 2 targeting serine residues (8, 9). Modification of serine residues strictly requires the formation of a PARP1/2:HPF1 complex, while its reversal is catalyzed by ARH3 (10, 11, 12, 13, 14). The linkage type defines the chemical and enzymatic stability of the ADP-ribosyl modification, thus influencing the signal dynamic and—depending on the modification context—may control signal recognition. Whether specific linkage types have defined regulatory functions, what drives the selection between different modification types, and how the modification dynamic is controlled are some of the open questions, especially for less abundant modification variants such as tyrosine ADP-ribosylation (Tyr-ADPr). Although consistently identified in ADP-ribosylomic data (15, 16, 17, 18, 19), Tyr-ADPr is among the least studied modification types: it has been suggested that some of the cellular Tyr-ADPr is synthesized by the PARP1:HPF1 complex (15, 19), but hydrolase(s) that cleave this modification remain elusive.

Here we present a detailed analysis of earlier ADP-ribosylome data and show that Tyr-ADPr is strongly linked with ribosome biogenesis, mRNA processing, and transcription repression and that Tyr-ADPr turn-over at some sites is dependent on ARH3. We established methodologies for the solid-phase synthesis of well-defined Tyr-ADPr peptides as well as their chemoenzymatic production. With these tools, we were able to show that the removal reaction is not only catalyzed by ARH3, which has been known as the main enzyme to remove Ser-ADPr (10), but also by PARG, which was thought to act primarily on ADP-ribose polymers (20, 21).

## Results

### Tyr-ADPr in pre-mRNA processing, transcription regulation, and ribosome biogenesis

**Figure 1:**
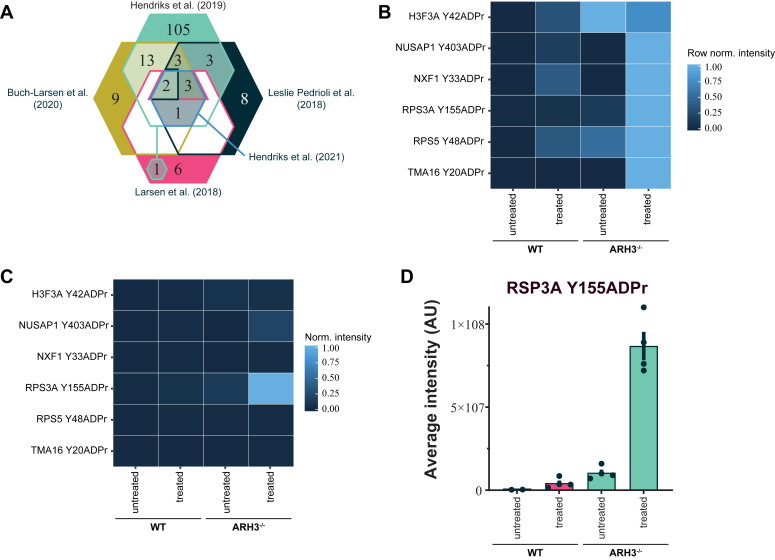
**Identification of Tyr-ADPr in ADP-ribosylome data.***A*, Euler diagram showing Tyr-ADPr site identification in recent publications (16, 17, 18, 19, 22). *B*, heatmap plot of average abundance for each Tyr-ADPr peptide identified in Hendriks *et al.* (17). Shown is the average intensity (*n* = 4 biological replicates) of each identified peptide normalized across experimental conditions. *C*, heatmap plot of average abundance for each Tyr-ADPr peptide (17) shown is the average intensity (*n* = 4 biological replicates) of each identified peptide normalized across all conditions. *D*, bar plot showing average abundance of RPS3A Y155ADPr identified in WT and ARH3^−/−^ cells untreated or treated with H_2_O_2_. Data is expressed as mean ± SEM of *n* = 4 biological replicates. Note, only two data points for WT untreated above the detection limit.

To gain first insight into the role of Tyr-ADPr, we re-analyzed previous ADP-ribosylomic data (16, 17, 18, 19, 22). In total, 128 proteins with 154 Tyr-ADPr sites were identified with a partial overlap between the studies (Fig. 1*A* and Table S1), but only ribosomal protein RSP3A was identified in all five datasets. Strikingly, of the identified proteins, 33 are involved in ribosomal biogenesis, 26 regulate transcription, 22 assist in pre-mRNA processing and translocation, and 15 in chromatin organization (Table S1). If the identified residues are of regulatory importance, one would expect them to be evolutionary conserved, hence we analyzed the 26 Tyr-ADPr sites (24 proteins) identified in more than one dataset for conservation across *Vertebrata* species with ZnF182 and a long form of RPL29 only present in *Mammalia* based on BLAST results. Most sites are highly conserved across species except for NCL, PPHLN1, and Y399 on NUSAP1 (Fig. S1 and Table S2). While our data do not support a strong sequential context for Tyr-ADPr, we saw enrichment of a Yx_0-1_[+] (100/154 sites) motifs (Fig. S1; (23)) in agreement with an earlier observation of a YK motif enrichment (18). The motif frequency is unaltered among the analyzed conserved sites (13/22 sites).

To further investigate the establishment and reversal of the Tyr-ADPr modification, we analyzed the large-scale proteome-wide data (17) focusing on the abundance of Tyr-ADPr peptides across the different experimental conditions: WT, ARH3^−/−^, and HPF1^−/−^ cells in the absence or presence of H_2_O_2_-induced DNA damage. No Tyr-ADPr sites were identified in HPF1^−/−^ cells, while ARH3^−/−^ cells showed a mild increase in peptide abundance (Fig. 1, *B* and *C*). Similarly, H_2_O_2_ induced a mild increase in Tyr-ADPr abundance in WT cells, while ARH3^−/−^ cells exhibited a pronounced increase in Tyr-ADPr, particularly for RSP3A (Fig. 1, *B*–*D*). These data are in line with earlier observations showing that recombinant PARP1:HPF1 can synthesize Tyr-ADPr *in vitro* (15, 19, 24).

### Chemical and enzymatic synthesis of Tyr-ADPr peptide

Since direct evidence for Tyr-ADPr reversal is as yet unavailable, we established the solid-phase synthesis of well-defined Tyr-ADP ribosylated peptides. The employed methodology was specifically modified to be compatible with the relatively acid-sensitive 4-*O*-tyrosyl ribofuranoside.

#### Building block synthesis

**Figure 2:**
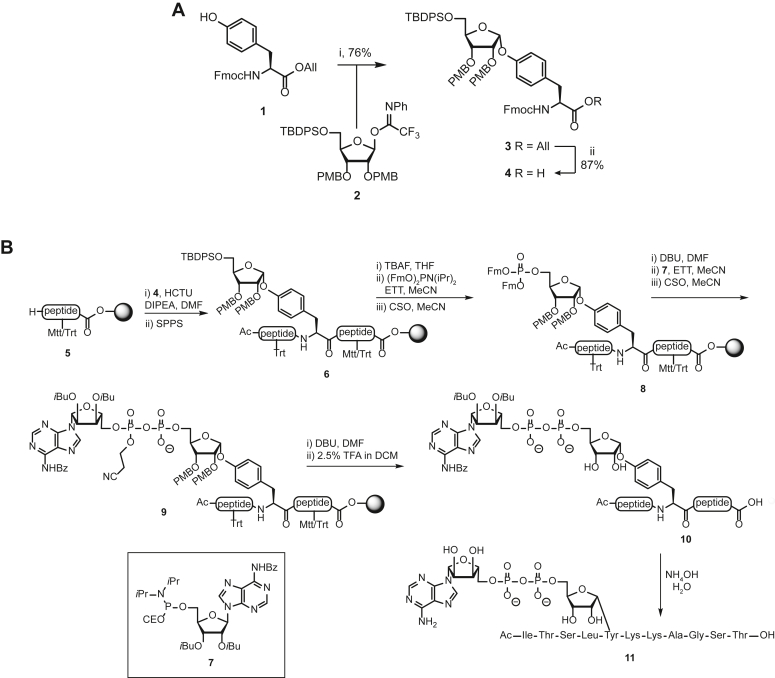
**Chemical synthesis of Tyr-ADPr peptide.***A*, scheme showing synthesis of Tyr-ribosylated building block 5 ready for SPPS. Reagents and conditions: (i) All-Br, DIPEA, DMF. (ii) 20% TFA in DCM, TIS. (iii) TBSOTf, DCM, −50 °C. (iv) Pd(PPh_3_)_4_, DMBA, DCM. *B*, scheme showing synthesis of Tyr-ADPr peptide 11 using acid-sensitive peptide segment protection and base-sensitive adenosine protective groups.

To acquire mono-Tyr-ADPr peptides, we initially employed our previous described synthetic strategy for serine, threonine, or cysteine MARylated peptides (25). First, we synthesized a ribosylated Tyr building block (4, Fig. 2*A*) to be used in Fmoc-based solid-phase peptide synthesis (Fmoc-SPPS). The suitably protected ribosyl acceptor Fmoc-Tyr(*t*Bu)-OAll 1 was prepared as described previously (26, 27), and the glycosylation reaction with ribosyl donor 2 was investigated (Fig. 2*A*), starting with the reported glycosylation conditions for the Ser, Thr, and Cys acceptors (Table 1, № 1; (25)).

**Table 1:** Optimization of the glycosylation conditions of acceptor 2 with donor 3

Entry №	Solvent	c(M)^a^	Activator	T(°C)	Reaction time (h)	Yield^b^
1	DCM	0.1	TMSOTf	−50	1	n.d.
2	DCM:dioxane (1:1)	0.1	TMSOTf	−50	1	n.d.
3	DCM	0.1	TMSOTf	−20	1	n.d.
4	DCM	0.03	TMSOTf	−50	1	38%
5	DCM	0.03	TBSOTf	−50	2.5	47%
6	DCM:dioxane (9:1)	0.03	TBSOTf	−50	2.5	67%

Abbreviation: DCM, dichloromethane.

a All reactions were carried out at a 0.2 mmol scale. c(M) is the concentration of the donor in the solvent with 0.1 equivalents of activator relative to the donor.

b n.d. = not determined.

Under these conditions, solubility issues arose as acceptor 1 did not dissolve properly in 0.1 M dichloromethane (DCM) at −50 °C. Therefore, a 1:1 mixture of DCM and 1,4-dioxane was tried (Table 1, № 2), which was shown to dissolve protected amino acids in a glycosylation reaction (28), but the use of this solvent mixture did not improve the solubility of the Tyr acceptor. Next, a solution of acceptor 1 and donor 2 in DCM at room temperature was prepared and remained clear during slowly cooling to −20 °C. Glycosylation was attempted at −20 °C (Table 1, № 3) as further cooling resulted in the precipitation of acceptor 1. However, activator addition resulted in a complex mixture of products that was inseparable by silica gel column chromatography. The formation of multiple products can be explained by the loss of α-stereoselectivity and the unwanted loss of the PMB-protecting groups under the acidic conditions of the glycosylation reaction at the −20 °C (29).

Decreasing the concentration of the reaction to 0.03 M allowed the temperature to be maintained at −50 °C without precipitation of acceptor 1 (Table 1, № 4). In this way, fully protected, ribosylated tyrosine building block 3 could be obtained in a moderate 38% yield (attributed to the slower reaction rate at the lower concentration). After increasing the reaction time to 2.5 h (Table 1, № 5) and switching to the milder activator TBSOTf, the yield of 3 increased to 47%. The addition of 10% (v/v) dioxane to the reaction mixture increased the solubility of the acceptor at −50 °C and improved the yield to 67% (Table 1, № 6). Scaling up the reaction to a 1 mmol scale increased the yield even further to 76% (Fig. 2*A*) and enough material was available to proceed with the synthesis of the required Fmoc-building block 4. Pd(0)-catalyzed cleavage of the allyl ester, using 1,3-diethyl barbituric acid as a scavenger, furnished orthogonally protected *O*-ribosylated Fmoc-tyrosine 4, suitable for the intended Fmoc-based SPPS strategy.

#### Solid phase synthesis of MARylated Tyr-peptide

After obtaining building block 4, we first attempted a solid-phase synthesis of peptide 11 (Fig. 2*B*) as previously described by us (Fig. S2; (29)). The LC-MS analysis of the crude Tyr-ADPr peptide 11 after final acidolytic deprotection with 10% TFA in DCM revealed a main product (∼75% based on the UV-absorption in the LC-MS trace) that did not correspond to target 11, but rather to the unmodified peptide. TFA-mediated acidolysis of the glycosidic bond of the tyrosyl ribofuranoside in 11 was suspected. Lowering the concentration of TFA in the cleavage cocktail to 5% did decrease the observed acidolysis to approximately 25% and reduction to 2.5% prevented the side reaction. Although the Trt, Mtt, and PMB groups were completely removed, the Boc protecting group used to protect the exocyclic adenine amine was found to be stable under these conditions and required ≥5% TFA for removal. To prevent the unwanted acidolysis, we opted for base-sensitive adenosine protecting groups (30) and used phosphoramidite 7 (Fig. 2*B*), where the 2′- and 3′-hydroxyl groups are protected with iso-butyryl while the exocyclic amine is masked with a benzoyl group. Standard elongation using Fmoc-SPPS and incorporation of Tyr-building block 4 led to immobilized peptide 6. TBAF mediated desilylation, followed by phosphitylation of the released alcohol with Fm-protected phosphoramidite and finally oxidation of the phosphite triester produced protected phosphotriester 8. Next, DBU mediated removal of the Fm groups in 8 yielded the corresponding phosphate monoester which was reacted with phosphoramidite 7. Oxidation of the resulting P^III^–P^V^ intermediate furnished immobilized, protected MARylated Tyr-peptide 9. Removal of the protecting groups started with DBU-mediated cyanoethyl elimination, followed by treatment with 2.5% TFA in DCM, inducing the removal of the Trt, Mtt, and PMB protecting groups and cleavage of the linker, to give partially protected peptide 10. Finally, the removal of all base labile adenosine protecting groups in 10 by treatment with a saturated, aqueous ammonium hydroxide solution resulted, after purification, in the isolation of Tyr-ADPr peptide 11 in 4.5% yield.

#### Chemoenzymatic synthesis of Tyr-ADPr by PARP1 and PARP2

**Figure 3:**
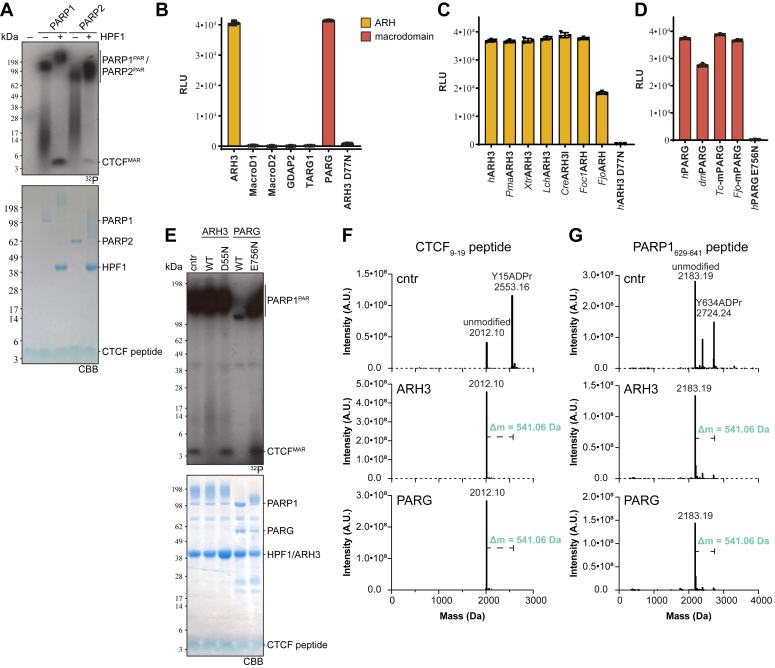
**ARH3 and PARG reverse HPF1-dependent Tyr-ADP ribosylation.***A*, CTCF_9-19_ (Table S3) was enzymatically modified by PARP1 or PARP2 in the presence or absence of HPF1. *B*, hydrolysis of Tyr-ADPr on peptide 11 by ARH3 and human macrodomains and detection by chemiluminescence using the AMP-Glo assay (Promega). Samples were measured in triplicates, background corrected, and represent mean ± SD. *C*, hydrolysis of peptide 11 by representatives of the ARH3-like family (*Parus major* [*Pma*ARH3], *Xenopus tropicalis* [*Xtr*ARH3], *Latimeria chalumnae* [*Lch*ARH3], *Chlamydomonas reinhardtii* [*Cre*ARH3-like], *Fusarium oxysporum* sp.f. *cubense* (race 1) [*Foc1*ARH], *Flavobacterium johnsoniae* [*Fjo*ARH]) and detection by AMP-Glo assay (Promega). Samples were measured in triplicates, background corrected, and represent mean ± SD. *D*, hydrolysis of peptide 11 by representatives of the PARG family (‘classical’ members *Homo sapiens* [*h*PARG] and *Drosophila melanogaster* [*dm*PARG], as well as microbial-type (mPARGs) *Thermomonospora curvata* [*Tc-m*PARG] and *Flavobacterium johnsoniae* [*Fjo*-mPARG]) and detection by AMP-Glo assay (Promega). Samples were measured in triplicates, background corrected, and represent mean ± SD. *E*, CTCF_9-19_ peptide was enzymatically modified by PARP1/HPF1. The modified peptide was demodified by either ARH3 or PARG using catalytically inactive mutants as controls. *F* and *G*, *top*: Deconvoluted MS spectrum of CTCF_9-19_ (*F*) and PARP1_629-641_ (*G*) mixtures of unmodified (masses = 2012.1 and 2183.19, respectively) and ADP-ribosylated peptides modified on Y15 (mass = 2553.16) and Y634 (mass = 2183.19), respectively. *Middle*/*Bottom*: Deconvoluted MS spectra of ADP-ribosylated peptides treated with 1 μM ARH3 (*middle*) or PARG (*bottom*) for 3 h at 37 °C. Mass of ADP-ribosyl moiety = 541.06 Da.

In addition to chemical synthesis, we applied an earlier described method for the enzymatic generation of Tyr-ADPr peptides (15, 24) to investigate whether Tyr-ADPr was carried out solely by PARP1 or if PARP2 can also establish the modification. We utilized the CTCF- and PARP1-derived model peptides containing Tyr-ADPr sites identified in different ADP-ribosylomic data (18, 19). We could demonstrate that both recombinant PARP1 and PARP2 readily modify the two peptides, but this activity was strictly dependent on HPF1 (Figs. 3*A* and S3*A*).

#### Tyrosine-ADP-ribosylation is reversed by ARH3 and PARG

Following establishment of both strategies to generate specific Tyr-ADPr–containing peptides, we were able to analyze whether recombinant ARH3 can hydrolyze this substrate as suggested by the data of Hendriks et al. (Fig. 1, *B*–*D*; (17)). Utilizing synthetic peptide 11 and our previously developed AMP-Glo-based assay (30, 31), we observed hydrolytic activity for WT human ARH3, but not its inactive mutant (D77N; Fig. 3*B*). Interestingly, this activity is conserved among evolutionary-related homologs from animals, fungi, plants, and bacteria (Fig. 3*C*). This includes previously uncharacterized distant homologous from the fungus *Fusarium oxysporum* f.sp. *cubense* (race 1; *Foc1*ARH) and the bacterium *Flavobacterium johnsoniae* (*Fjo*ARH). For comparison, we analyzed hydrolytic activity of several human macrodomains (Fig. 3*B*). Strikingly, we observed highly efficient cleavage of Tyr-ADPr by PARG (Fig. 3*B*). To confirm this, we probed homologs from *Drosophila* (*human-like PARG domain*: *IPR046372)*, which was shown to act as mono-Ser-ADPr hydrolase (32), and two bacterial species (*Thermomonospora curvata* [*Tc*PARG] (21) and *F. johnsoniae* [*Fjo*-mPARG; microbial PARG domain: IPR019261]) and observed activity for all homologs (Fig. 3*D*).

To exclude the possibility that sequence context of the modification influences the hydrolase reaction, we tested the known Tyr-ADP-ribosylatable peptides from CTCF, PARP1, and histone H3 (Table S3) as substrate for ARH3 and PARG and found that both enzymes could reverse the modification irrespective of the surrounding sequence (Figs. 3, *E*–*G* and S3*B*).

## Discussion

ADP-ribosylation is intimately linked with cellular stress responses such as oxidative stress, disease conditions, and bacterial and viral infections (3, 33). One of the major mechanisms to regulate activities of different ARTs is modification reversal applying to both protein and nucleic acid modifications (1, 5) as well as accessory factors, *for example,* formation of Ser-ADPr requires the PARP1/2:HPF1 complex (10, 12, 13). Our analysis of available ADP-ribosylome data (16, 18, 19) shows that among the many functions of PARP1/2, mRNA processing and ribosome biogenesis appear to be tightly linked to Tyr-ADPr. Both processes ultimately regulate protein translation and thus may influence adaptation to cellular stresses. This corresponds well with earlier observations showing that PARP1 can influence pre-ribosome assembly and cause rDNA silencing and mRNA maturation steps such as poly(A) tail establishment (7, 34, 35, 36, 37). PARP1 was shown to suppress poly(A) polymerase activity under heat shock conditions, thus controlling the maturation of pre-mRNAs (34). In this context, it is interesting to note that the Tyr-ADPr site of PABP2 (Table S1) is located in the N terminus with close proximity to the poly(A) polymerase interaction motif (38), thus allowing speculation that Tyr-ADPr modulates this interaction. Similarly, several Tyr-ADPr sites identified on ribosomal proteins are in the ribosome interior and thus being (i) inaccessible for modification and (ii) sterically clash with other ribose components. This suggests that Tyr-ADPr of these components could prevent assembly into ribosomes, hence slowing ribosome biogenesis. Noteworthy, the ADP-ribosylomic studies identifying Tyr-ADPr sites used oxidative stress as a cellular stimulant, which is known to negatively impact both transcription and translation (3). Additionally, earlier studies showed that PARP1 can also be activated by snoRNAs (39), thus providing a possibility for PARP1 to influence ribosome biogenesis in the absence of DNA damage. However, we cannot exclude the possibility that Tyr-ADPr can be synthesized also by some other PARP family members.

However, the study of these processes requires tools to separate the role of Tyr-ADPr from other modifications and to understand more deeply its spatiotemporal dynamic. The synthesis of well-defined Tyr-ADPr–modified peptides presented here provides just such a tool and will allow detailed analysis of potential Tyr-ADPr readers. We already utilized the peptides to show that both ARH3 and PARG can remove the Tyr-ADPr modification *in vitro*, thus supporting our finding that ARH3 affects Tyr-ADPr *in vivo*. The activity of PARG on peptide 11 is intriguing as in earlier studies, we were unable to observe activity against a near identical peptide in which the Tyr-ADPr was replaced with a Ser-ADPr site (29). This indicates that human PARG can discriminate between MARylation on tyrosine and serine residues. This is different from *Drosophila* PARG, which evolved subtle changes in its active site to allow cleavage of both Ser- and Tyr-ADPr (Fig. 3*E*; (32)). This adaptation is probably a consequence of the secondary loss of ARH3 in *Drosophila* lineages (32). Further diversification of PARG activity was observed for Glu-ADPr on which human PARG exhibits only low activity *in vitro*, whereas significant activity was observed for a plant homolog (40, 41, 42). However, in cellular contexts, other factors may increase the activity of human PARG towards Asp/Glu-mono-ADPr given that it has been recently shown that PARG inhibition causes a clear increase in the cellular levels of Asp/Glu-mono- and poly-ADPr (41, 43). Observed activity of bacterial PARGs and ARHs could indicate existence of Tyr-ADPr in bacteria, but there have been as yet no bacterial ADP-ribosylomics studies that tried to address this question.

In addition to the intriguing biology implications, our study also highlights technical challenges and opens new methodological opportunities. Synthetic peptides can greatly assist in elucidating the physiological role of Tyr-ADPr as demonstrated for peptides with other ADP-ribosylated amino acids (29, 30, 42, 44, 45, 46) and, *for example,* may provide a tool to study the readers of the DNA damage response ADP-ribosylome, similarly to what has been done recently with Ser-ADPr peptides (47). Combining the ADP-ribosylated peptides with biotin or bioorthogonal (“click chemistry”) tags will allow their utilization as bait in pull-down studies for cellular interactomes. Moreover, the discovery of ARH3 and PARG as efficient Tyr-ADPr hydrolases (Figs. 3 and S3) has important implications for identifying Tyr-ADPr sites *in vivo*. PARG is routinely used in ADP-ribosylome MS analyses to trim poly-ADP-ribose chains (16, 48, 49), therefore it is likely that most Tyr-ADPr is removed during MS sample processing, which thus may indicate a method-based underappreciation in the available ADP-ribosylomics datasets. We, therefore, suggest a re-evaluation of protocols involving PARG in the hope that this will eliminate this potential problem, leading to a more complete appreciation of the cellular Tyr-ADP-ribosylome (50) and support the discovery of new physiological roles of ADP-ribosylation signaling.

## Experimental procedures

### Detection of (ADP-ribosyl)hydrolase activity by AMP-Glo assay

The assay was performed as previously described (29, 30, 31). Briefly, 8 μM peptide was demodified by incubation with 1 μM indicated hydrolase for 30 min at 30 °C in assay buffer (50 mM Tris–HCl [pH 8], 200 mM NaCl, 10 mM MgCl_2_, 1 mM DTT, and 0.2 μM human NudT5). Reactions were stopped and analyzed by performing the AMP-Glo assay (Promega) according to the manufacturer’s protocol. Luminescence was recorded on a SpectraMax M5 plate reader (Molecular Devices) and data were analyzed with GraphPad Prism 10.2. For background, subtraction reaction were carried out in the absence of hydrolase.

### PARG and ARH3 treatment of Tyr-ADPr peptides

The purified Tyr-ADPr peptides were resuspended in PARP reaction buffer (50 mM Tris–HCl [pH 7.5], 50 mM NaCl, 1 mM MgCl_2_) then incubated with or without 1 μM of PARG or ARH3 for 3 h at 37 °C. Next, the reaction was stopped by the addition of 2% FA, and the peptides were desalted and purified with StageTips. Each reaction was performed twice independently with comparable results.

### Radioenzymatic (de)modification assays of Tyr-peptides

Modification of target peptides by PARP1 and PARP2 was carried out as described earlier (12). Briefly, 0.5 μg peptide (Table S3) were modified in ADPr buffer (50 mM Tris–HCl [pH 8], 100 mM NaCl, 1 mM MgCl_2_) containing 1 μM PARP1 or PARP2, 15 mM NAD^+^, 0.5 μCi ^32^P-NAD^+^ (Hartmann Analytic), and 1 μM activated DNA duplex (Table S4) in the absence of presence of 2 μM HPF1 for 2 h at 37 °C. Reaction were stopped by the addition of 4× LDS buffer and analyzed by SDS-PAGE and autoradiography.

For demodification assay, the above reactions were stopped by the addition of 1 μM olaparib and demodification were carried out by the addition of 1 μM indicated hydrolase and further incubation for 1 h at 37 °C. Reactions were stopped by the addition of 4× LDS buffer and analyzed by SDS-PAGE and autoradiography.

### Re-analysis of Hendriks *et al.*

Raw data files and resulting MaxQuant analysis originally generated by Michael Nielsen’s lab (17; Data S6) were filtered for high confidence Tyr-ADPr sites using R programming language (v4.2.2). The average intensities of the six identified sites for each condition (WT treated/untreated and ARH3^−/−^ treated/untreated) were extracted and plotted as normalized heatmaps: either across tested conditions or across the whole Tyr-ADPr dataset. For bar plot of RPS3A Y155, the average intensity ± SEM of n = 4 replicates was plotted.

### Tyr-ADPr sites conservation analysis

Sequences for Tyr-ADPr site conservation analysis were identified by BLASTp search using the human protein sequence as input (Table S2). Full-length sequences were aligned in JalView (v 2.11.3.2) using MAFFT L-INS-i (51, 52). Sequences around the Tyr-ADPr site were exported into ALINE for visualization (53).

## Data availability

The genomic sequence of *Foc1*ARH was deposited in GenBank under the accession number PP747355.

## Supporting information

This article contains supporting information (10, 11, 12, 14, 15, 24, 26, 28, 32, 54, 55, 56).

## Conflict of interest

E. P. is an employee of Vertex Pharmaceuticals and may own stock or stock options in that company.
