# Supplementary material for: Reversal of tyrosine-linked ADP-ribosylation by ARH3 and PARG
Source: J Biol Chem. 2024 Sep 27;300(11):107838. doi: 10.1016/j.jbc.2024.107838 (PMC11541775; doi:10.1016/j.jbc.2024.107838)
Supplement: Supporting Information [file mmc1.pdf]

Supporting Information

Reversal of tyrosine-linked ADP-ribosylation by ARH3 and PARG

Johannes Gregor Matthias Rack[1,‡,¶], Jim Voorneveld[2,‡], Edoardo José Longarini[3,4], Sven Wijngaarden[2], Kang Zhu[5,6], Alessandra Peters[5], Jia Jhing Sia[5,7], Evgeniia Prokhorova[5], Dragana Ahel[5], Ivan Matić [3,8], Dmitri V. Filippov[2,¶], Ivan Ahel[5,¶]

[1]MRC Centre for Medical Mycology, University of Exeter, Geoffrey Pope Building, Stocker Road, Exeter, EX4 4QD, UK

[2]Leiden Institute of Chemistry, Leiden University, Einsteinweg 55, 2333 CC Leiden, The Netherlands

[3]Research Group of Proteomics and ADP-ribosylation Signalling, Max Planck Institute for Biology of Ageing, 50931 Cologne, Germany.

[4]Present address: Department of Chemistry, Princeton University, Princeton, NJ, USA

[5]Sir William Dunn School of Pathology, University of Oxford, South Parks Road, Oxford, OX1 3RE, UK

[6]Present address: Health Science Centre, East China Normal University, Shanghai, 200241, China

[7]Current address: Cambridge Institute of Therapeutic Immunology & Infectious Disease (CITIID), Jeffrey Cheah Biomedical Centre, Department of Medicine, University of Cambridge, Cambridge, CB2 0AW, UK.

[8]Cologne Excellence Cluster for Stress Responses in Ageing-Associated Diseases (CECAD), University of Cologne, 50931 Cologne, Germany

‡These authors contributed equally to this work.

¶**Correspondence to:** Johannes G. M. Rack, Dmitri V. Filippov, and Ivan Ahel

Supplemental experimental procedure

General synthetic procedures for Tyr-ADPr peptide synthesis

All reagents were used as received unless stated otherwise. Solvents used in synthesis were dried and stored over 4 Å molecular sieves, except for MeOH and MeCN which were stored over 3 Å molecular sieves. Triethylamine (TEA) and diisopropylethylamine (DIPEA) were stored over KOH pellets. Column chromatography was performed on silica gel 60 Å (40-63 µm, Macherey-Nagel). TLC analysis was performed on Macherey-Nagel aluminium sheets (silica gel 60 F_{254}). TLC was used to visualize compounds by UV at wavelength 254 nm and by spraying with either cerium molybdate spray (25 g/L $(NH_4)_6Mo_7O_{24}$, 10 g/L $(NH_4)_4Ce(SO_4)_4 \cdot H_2O$ in 10% H_2SO_4 water solution) or $KMnO_4$ spray (20 g/L $KMnO_4$ and 10 g/L K_2CO_3 in water) followed by charring at approx. 250 °C. LC-MS analysis was performed on a Finnigan Surveyor HPLC system with a Nucleodur C18 Gravity 3 µm 50 x 4.60 mm column (detection at 200-600 nm) coupled to a Finnigan LCQ Advantage Max mass spectrometer with ESI or coupled to a Thermo LCQ Fleet Ion mass spectrometer with ESI. The method used was 10→90% 13.5 min (0→0.5 min: 10% MeCN; 0.5→8.5 min: 10% to 90% MeCN; 8.5→ 11 min: 90% MeCN; 11→13.5 min: 10% MeCN) or 0→50% 13.5 min. NMR spectra were recorded on a Bruker AV-400, AV-500 or AV-600 instrument. Chemical shifts (δ) are given in ppm relative to tetramethyl silane as internal standard. Coupling constants (*J*) are given in Hz. For compounds **11** and **15**, a small amount of EDTA was added to the NMR sample to sharpen the peaks for ^{31}P-NMR. All given ^{13}C-APT spectra are proton decoupled.

1-*O*-(2,3-bis-*O*-(4-methoxybenzyl)-5-*O*-((*tert*-butyl)-diphenylsilyl)-α-D-ribosyl)-*N*-fluorenylmethoxycarbonyltyrosine allyl ester (3)

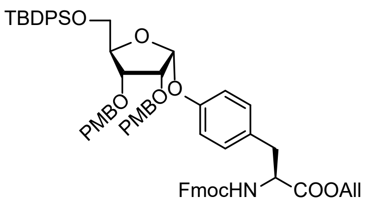

Compound **2** (1.10 g, 1.375 mmol, 1.1 eq.; (1)) and Fmoc-Tyr(OH)-OAll **1** (554 mg, 1.25 mmol, 1.0 eq. relative to the donor; (2)) were co-evaporated thrice with toluene and dissolved in DCM:dioxane (9:1, 46 mL, 0.03 M donor concentration). The reaction was cooled to -50 °C and TBSOTf (29 µL, 0.14 mmol, 0.1 eq. relative to the donor) was added. The reaction was stirred at -50 °C flor 2.5 hours after which TLC analysis showed near full conversion of the starting material. The reaction was quenched with TEA and concentrated *in vacuo*. Flash column chromatography (0.5→3% acetone in DCM) yielded the title compound as a clear oil (1005 mg, 0.953 mmol, 76%). **Rf:** 0.58 in 3% acetone in DCM. **^{1}H NMR:** (400 MHz, $CDCl_3$) δ 7.78 – 7.70 (m, 2H, Fmoc arom.), 7.67 – 7.51 (m, 6H, Fmoc arom. + TBDPS arom.), 7.48 – 7.24 (m, 14H, Fmoc arom. + TBDPS arom. + PMB arom.), 7.11 – 6.97 (m, 4H, Tyr arom.), 6.86 – 6.75 (m, 4H, PMB arom.), 5.88 (ddd, *J* = 16.5, 10.6, 5.2 Hz, 1H, $CH_2C\mathit{H}CH_2$), 5.51 (d, *J* = 4.3 Hz, 1H, H-1), 5.38 – 5.19 (m, 3H, NH + $CH_2CHC\mathit{H}_2$), 4.74 – 4.50 (m, 8H, CH Ser + $C\mathit{H}_2CHCH_2$), 4.44 (dd, *J* = 10.6, 7.1 Hz, 1H, CH_{2a} Fmoc), 4.40 – 4.29 (m, 1H, CH_{2b} Fmoc), 4.29 – 4.15 (m, 2H, H-4 + CH Fmoc), 4.09 (dd, *J* = 6.5, 2.8 Hz, 1H, H-3), 3.99 (dd, *J* = 6.4, 4.3 Hz, 1H, H-2), 3.78 (s, 3H, CH_3 PMB), 3.76 (s, 3H, CH_3 PMB), 3.58 (ddd, *J* = 53.4, 11.3, 3.1 Hz, 2H, H-5), 3.10 (d, *J* = 5.7 Hz, 2H, CH_2 Tyr), 0.95 (s, 9H, *t*Bu TBDPS). **^{13}C NMR:** (101 MHz, $CDCl_3$) δ 171.3 (C=O COOAll), 159.4, 159.3 (Cq PMB), 156.9 (C=O Fmoc), 155.7 (Cq Tyr), 143.9, 143.8, 141.4 (Cq Fmoc), 135.7, 135.6 (CH arom. TBDPS), 133.2, 133.1 (Cq TBDPS), 131.5 ($CH\mathit{C}HCH_2$), 130.4 (Cq PMB), 130.3 (CH arom. Tyr), 129.9, 129.8, 129.7, 129.7 (CH arom.), 128.9 (Cq PMB), 127.9, 127.9, 127.8, 127.8, 127.8, 127.2, 125.3, 125.2 (CH arom.), 120.1, 120.0 (CH arom. Fmoc), 119.3 ($CH_2CH\mathit{C}$, 117.5 (CH arom. Tyr), 113.9, 113.9, 113.8 (CH arom. PMB), 100.0 (C-1), 84.2 (C-4), 77.7 (C-2), 75.0 (C-3), 72.4, 72.1 (CH_2 PMB), 67.1 (CH_2 Fmoc), 66.2 ($\mathit{C}H_2CHCH_2$), 64.0 (C-5), 55.3, 55.3 (CH_3 PMB), 54.9 (CH Tyr), 47.2 (CH Fmoc), 37.5 (CH_2 Tyr), 26.9 (CH_3 *t*Bu), 19.3 (Cq *t*Bu). **HRMS:** $[C_{64}H_{67}NO_{11}Si + Na]^+$ found: 1076.4374, calculated: 1076.4376.

1-*O*-(2,3-bis-*O*-(4-methoxybenzyl)-5-*O*-((*tert*-butyl)-diphenylsilyl)-α-D-ribosyl)-*N*-fluorenylmethoxycarbonyl tyrosine (4)

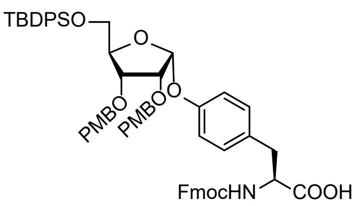

Compound **3** (626 mg, 0.594 mmol, 1.0 eq.) was dissolved in DCM (6.0 mL, 0.1 M). DMBA (184 mg, 1.18 mmol, 2.0 eq.) and $Pd(PPh_3)_4$ (6.9 mg, 5.9 µmol, 0.01 eq.) were added and the reaction was stirred for 1 hour before TLC showed full conversion of the starting material into a lower running product. The reaction was diluted with DCM, washed with 1 M HCl and the organic layer was dried over $MgSO_4$, filtered, and concentrated *in vacuo*. Flash column chromatography in (4→6% MeOH in DCM) yielded the title compound as a white foam (518 mg, 0.511 mmol, 87%). **Rf:** 0.50 in 5% MeOH in DCM + 0.1% AcOH. **^{1}H NMR:** (400 MHz, $CDCl_3$) δ 7.72 (d, *J* = 7.5 Hz, 2H, Fmoc arom.), 7.65 – 7.49 (m, 6H, Fmoc arom. + TBDPS arom.), 7.47 – 7.20 (m, 14H, Fmoc arom. + TBDPS arom. + PMB arom.), 7.09 – 6.97 (m, 4H, Tyr arom.), 6.88 – 6.75 (m, 4H, PMB arom.), 5.48 (d, *J* = 4.3 Hz, 1H, H-1), 5.32 (d, *J* = 8.1 Hz, 1H, NH), 4.70 – 4.51 (m, 5H, 2x CH_2 PMB + CH Ser), 4.44 (dd, *J* = 10.5, 7.2 Hz, 1H, CH_{2a} Fmoc), 4.32 (dd, *J* = 10.8, 7.1 Hz, 1H, CH_{2b} Fmoc), 4.28 – 4.15 (m, 2H, H-4 + CH Fmoc), 4.08 (dd, *J* = 6.5, 2.6 Hz, 1H, H-3), 3.97 (dd, *J* = 6.5, 4.4 Hz, 1H, H-2), 3.76 (s, 3H, CH_3 PMB), 3.74 (s, 3H, CH_3 PMB), 3.63 (dd, *J* = 11.1, 3.3 Hz, 1H, H-5$_a$), 3.49 (dd, *J* = 11.2, 2.8 Hz, 1H, H-5$_b$), 3.18 – 3.01 (m, 2H, CH_2 Tyr), 0.94 (s, 9H, *t*Bu TBDPS). **^{13}C NMR:** (101 MHz, $CDCl_3$) δ 174.8 (C=O COOH), 159.4, 159.3 (Cq PMB), 156.8 (C=O Fmoc), 155.9 (Cq Tyr), 143.9 9, 143.8, 141.4 (Cq Fmoc), 135.7, 135.6 (CH arom. TBDPS), 133.2, 133.1 (Cq TBDPS), 130.4 (CH arom. Tyr), 130.2 (Cq PMB), 129.9, 129.9, 129.8, 129.8, 129.6 (CH arom.), 129.0 (Cq PMB), 127.9, 127.9, 127.8, 127.2, 125.3, 125.2, 120.0 (CH arom.), 117.5 (CH arom. Tyr), 114.0, 114.0, 113.8 (CH arom. PMB), 99.8 (C-1), 84.3 (C-4), 77.8 (C-2), 75.0 (C-3), 72.4, 72.1 (CH_2 PMB), 67.2 (CH_2 Fmoc), 64.0 (C-5), 55.3 (CH_3 PMB), 54.7 (CH Tyr), 47.2 (CH Fmoc), 37.0, (CH_2 Tyr), 26.9 (CH_3 *t*Bu), 19.3 (Cq *t*Bu). **HRMS:** $[C_{61}H_{63}NO_{11}Si + Na]^+$ found: 1036.4060, calculated: 1036.4063.

General procedures for solid phase synthesis

Peptide synthesis

The intermediate peptides were synthesized using standard, Fmoc-based solid phase peptide synthesis utilizing (pre-loaded) TentaGel® S AC purchased from Rapp Polymer GmbH. Coupling cycles were as followed: Fmoc deprotection: 2x2 minutes, 1x5 minutes treatment with 20% piperidine in DMF. Coupling: treatment of 6 eq. amino acid, 6 eq. HCTU (0.25 M in DMF) and 12 eq. DIPEA (1 M in DMF) for 30 minutes. Capping: 2x2 minutes treatment of the resin with a 10% Ac_2O solution in DMF and catalytic DIPEA. Washing between the steps was done with DMF. Ribosylated amino acid **5** was incorporated in the sequence by adding a solution of 2 eq. building block in a 0.25 M HCTU solution (2 eq.) in DMF and a 1 M DIPEA solution (4 eq.) in DMF to the resin in a fritted syringe. The resin was shaken overnight and thoroughly washed.

On-resin phosphorylation

The resin was treated with a sufficient amount of 1 M TBAF in THF (enough so that the entirety of the resin is submerged) for 30 minutes. The resin was thoroughly washed with DCM and DMF before the treatment was repeated once, furnishing the desilylated intermediate. The resin was then extensively washed with MeCN and flushed with nitrogen to remove traces of water before the resin was subjected to a solution of 5 eq. of $(FmO)_2PN(iPr)_2$ (0.25 M in MeCN) with 10 eq. ETT solution (0.25 M in MeCN). The resin was shaken for 30 minutes after which the resin was washed with MeCN. The resin was then treated with a sufficient amount of CSO solution (0.5 M in MeCN) for 30 minutes. The resin was then treated with a 10% DBU solution in DMF (2x 15 minutes) to furnish the crude, immobilized and deprotected phosphoribosylated peptide.

Construction of the pyrophosphate

The resin was extensively washed with MeCN and flushed with nitrogen to remove traces of water. The resin was then treated with a solution of compound **12** (3 eq., 0.3 M in MeCN) and ETT (6 eq., 0.25 M in MeCN) for 30 minutes. The resin was thoroughly washed with MeCN before a sufficient amount of CSO (0.5 M in MeCN) was added to the resin and shaken for 30 minutes.

Final deprotection and cleavage

The resin was then treated with a 10% DBU solution in DMF (2x 10 minutes) to remove the cyano ethyl protecting group. The resin was then treated with a 1 M TBAF solution in THF (2x 45 minutes) and washed with DMF followed by DCM. Final cleavage/deprotection occurred by treating the resin with a cleavage cocktail (2.5:2.5:95 TIS:TFA:DCM) for 1 hour. The crude products were collected by filtration and the resin was washed with a solution of 1:1:1 water:*t*BuOH:MeCN. The solvents were evaporated *in vacuo* and co-evaporated with a 1:1:1 water:*t*BuOH:MeCN solution.

Ac-Ile-Thr-Ser-Leu-Tyr(5-*O*-adenosine-diphosphate-α-D-ribosyl)-Lys-Lys-Ala-Gly-Ser-Thr-OH (11)

50 µmol TentaGel® S AC resin was loaded by treating the resin with 2.5 mL of a 0.2 M Fmoc-Thr(Trt)-OH solution (10 eq.) and DIC (77 µL, 0.5 mmol, 10 eq.) in DMF together with a catalytic amount of DMAP for 2 hours after which the general procedures described above were applied to the resin. The amino acids used were: Fmoc-Ile-OH, Fmoc-Thr(Trt)-OH), Fmoc-Ser(Trt)-OH, Fmoc-Leu-OH, Fmoc-Lys(Mtt)-OH, Fmoc-Ala-OH, Fmoc-Gly-OH and **4**. The crude peptide was purified by RP-HPLC in NH_4OAc buffer. The pure fractions were concentrated, co-evaporated extensively with a 1:1 mixture of MeCN:Milli-Q water, redissolved in Milli-Q water and lyophilized to obtain the title compound as a white solid (3.98 mg, 2.28 µmol, 4.5%). **^{1}H NMR:** (500 MHz, D_2O) δ 8.35 (s, 1H, H-2 adenine), 8.10 (s, 1H, H-8 adenine), 6.88 (d, J = 8.5 Hz, 2H, Tyr arom.), 6.74 (d, J = 8.5 Hz, 2H, Tyr arom.), 5.97 (d, J = 5.5 Hz, 1H, H-1' adenosine), 5.38 (d, J = 4.5 Hz, 1H, H-1' ribosyl). **^{31}P NMR:** (202 MHz, D_2O) δ -11.1, -11.2, -11.3, -11.4. **LC-MS:** (0 -> 50% B in A) Rt = 5.87. **HRMS:** $[C_{69}H_{112}N_{18}O_{31}P_2 + 2H]^{2+}$ found: 876.3677, calculated: 876.3681.

Plasmid construction

Flavobacterium johnsoniae (NCIMB 11054) was grown on Anacker-Ordal Medium (0.5 g/L pancreatic digest of casein, 0.5 g/L yeast extract, 0.2 g/L beef extract, 0.2 g/L sodium acetate, and 11 g/L agar) and genomic DNA extracted using the DNeasy UltraClean Microbial Kit (Qiagen). Coding sequence of the (ADP-ribosyl)hydrolase (*Fjo*ARH; WQG79355) was amplified by standard PCR and transferred into $pET9H_3$ (3) via NcoI/BamHI restriction sites in frame with the N-terminal HRV3C cleavage site and His-tag. Microbial-type PARG (*Fjo*-mParg; ABQ03789) was amplified by standard PCR using a forward primer with HRV3C cleavage sequence in frame with *Fjo*-mParg and His-tag and transferred into pDEST17 via Gateway cloning (Invitrogen).

ARH3-like from *Chlamydomonas reinhardtii* (*Cre*ARH3l; XP_001698094) was cloned from cDNA via standard PCR amplification. The ORF was inserted in pET21a(+) (Novagen) via NdeI/XhoI restriction sites and is in frame with the C-terminal His-tag.

Genomic DNA from *Fusarium oxysporum* sp.f. *cubense* race 1 (strain IMI141109) was obtained from CABI (Wallingford, UK). The genomic region encoding the (ADP-ribosyl)hydrolase (*Foc1*ARH) was amplified by standard PCR and transferred into pJET1.2 cloning vector (Thermo Fisher Scientific) and sequence was verified by Sanger sequencing of three independent clones. The ORF was amplified by standard PCR and inserted into $pET9H_3$ (3) via NcoI/BamHI restriction sites in frame with the N-terminal HRV3C cleavage site and His-tag.

All indicated mutations were introduced via PCR-based site-directed mutagenesis using QuikChange Lightning Site-Directed Mutagenesis Kit (Agilent Technologies) according to manufacturer's instructions.

Expression constructs of PARP1, PARP2, PARG, GDAP2, MacroD1, MacroD2, TARG1, *dm*PARG, *Tc*PARG, HPF1, ARH3, *Pma*ARH3, *Xtr*ARH3, and *Lch*ARH3 were described before (4-10).

Protein Expression

Expression and purification of PARP1, PARP2, PARG, GDAP2, MacroD1, MacroD2, TARG1, *dm*PARG, HPF1 and ARH3 were described before (5-10). All other recombinant proteins were expressed in Rosetta (DE3) cells grown in LB medium supplemented with 2 mM $MgSO_4$ and appropriate antibiotics at 37 °C to OD_{600} 0.6. Expression was induced with 0.4 mM IPTG and cells were grown at 17 °C overnight before harvested by centrifugation. Recombinant His-tagged proteins were purified by Ni^{2+}-NTA chromatography (Jena Bioscience) according to the manufacturer's protocol using the following buffers: all buffer contained 50 mM TrisHCl [pH 8] and 500 mM NaCl; additionally, the lysis/binding buffer contained 25 mM imidazole, the washing buffer contained 50 mM imidazole, and the elution buffer contained 500 mM imidazole. Proteins were dialyzed over night at 4 °C against 50 mM TrisHCl [pH 8], 200 mM NaCl, 1 mM DTT, 5% (v/v) glycerol and stored at -80 °C until use.

Chemoenzymatic synthesis of Tyr-ADPr peptides for MS assays

Tyr-ADPr peptides were prepared as previously described (11). Briefly, 621 µM of the respective unmodified peptides (Tab. S3) were incubated with 0.1 µM PARP1, 5 µM HPF1, 1 µg/µL sonicated DNA, and 2 mM NAD^+ in PARP reaction buffer (50 mM TrisHCl [pH 7.5], 50 mM NaCl, 1 mM $MgCl_2$) for 6 h at RT, with addition of 2 mM NAD^+ every 2 h. Even under these optimized reaction conditions, the HPF1/PARP1 chemoenzymatic installation of Tyr-ADPr results in low ADPr yield. To purify the resulting Tyr-ADPr peptides, we used boronic acid purification. The peptides were diluted with 100 mM ammonium bicarbonate (ABC, [pH 9]) and incubated for 1 h with boronic acid agarose beads (Sigma-Aldrich). The peptide-beads suspension was transferred on top of a micro spin column, washed five times with 1 M ABC [pH 9], two times with 100 mM ABC [pH 9], then eluted three times with 1% FA. The eluate was dried with a speedvac. Dried peptides were resuspended in 100 mM ABC [pH 9] and desalted with StageTips.

LC-MS/MS analysis of Tyr-ADPr peptides

Analysis of Tyr-ADPr peptides, before or after enzymatic reactions, was performed as previously described (11). Briefly, liquid chromatography for all LC-MS/MS runs was performed on an EASY-nLC 1200 Liquid Chromatography system (Thermo Scientific) coupled to the spectrometers via modified NanoFlex sources (Thermo Scientific). Peptides were loaded onto 250-mm x 75-µm PicoFrit (C18 2 µm medium) analytical columns at a maximum pressure of 800 bar. Solutions A and B for the UPLCs were 0.1% FA in water and 80% ACN, respectively. Samples were loaded in 0.1% FA in water to maximize retention of highly hydrophilic peptides. 19-minute gradients were: 0:00 to 13:00 min (0% B to 31% B); 13:00 to 16:20 min (31% B to 44% B) and 16:20 to 19:00 min (44% B to 95% B).

Supplemental Tables

Table S3. Commercial synthetic peptides used in this study.

Peptide	Position in protein[a]	Sequence[b]
CTCF	9-19	LVRHRRYKHTHGGK
H3	33-51	TGGVKKPHRYRPGTVALRGGK
PARP1	629-641	KNFTKYPKKFYPLGGK

(a) Corresponding to human protein
(b) Peptides carried the following modifications: N-terminal acetylation, C-terminal amidation, and biotinylation on the C-terminal lysine

Table S4. Activating DNA sequences.

name	Sequence (5'→3')
aDNA_for	ATCAGATAGCATCTGTGCGGCCGCTTAGGG
aDNA_rev	CCCTAAGCGGCCGCACAGATGCTATCTGAT

Supplemental Figure Legends

Figure S1. Tyr-ADPr site conservation amongst *Vertebrata*.
Multiple sequence alignment of selected protein from indicated *Vertebrata* species. Full-length sequences (Tab. S2) were aligned with MAFFT L-INS-i and regions surround the Tyr-ADPr modification site (Tab. S1) isolated for visualisation. Tyr-ADPr sites are indicated by Ҁ symbol and number of reporting publication is given in brackets. (*) Also identified in (12), (¶) modification is located on a C-terminal extension only found in *Mammalia*, and (π) ZnF182 was only identified in *Mammalia*.

Figure S2. Originally attempted synthesis strategy of Tyr-ADPr peptide 11.

Figure S3. Tyr-ADPr establishment and reversal.
(A) PARP1$_{629\text{-}641}$ peptide (Tab. S3) was enzymatically modified by PARP1 or PARP2 in presence or absence of HPF1.
(B) Top: deconvoluted MS spectrum of H3$_{33\text{-}51}$ peptide ADPribosylated on Y42 (mass = 3043.46). Bottom: deconvoluted MS spectrum of H3$_{33\text{-}51}$Y42ADPr treated with 1 µM PARG for 3 h at 37 ˚C. The in vitro PARG reaction results in a delta mass of 541.06 Da, corresponding to the loss of the ADPr moiety.

Supplemental References

1. Kistemaker, H. A., van Noort, G. J., Overkleeft, H. S., van der Marel, G. A., and Filippov, D. V. (2013) Stereoselective ribosylation of amino acids Org Lett **15**, 2306-2309 10.1021/ol400929c
2. van der Heden van Noort, G. J., Overkleeft, H. S., van der Marel, G. A., and Filippov, D. V. (2010) Synthesis of nucleotidylated poliovirus VPg proteins J Org Chem **75**, 5733-5736 10.1021/jo100757t
3. Rack, J. G., Morra, R., Barkauskaite, E., Kraehenbuehl, R., Ariza, A., Qu, Y. *et al.* (2015) Identification of a Class of Protein ADP-Ribosylating Sirtuins in Microbial Pathogens Mol Cell **59**, 309-320 10.1016/j.molcel.2015.06.013
4. Fontana, P., Bonfiglio, J. J., Palazzo, L., Bartlett, E., Matic, I., and Ahel, I. (2017) Serine ADP-ribosylation reversal by the hydrolase ARH3 Elife **6**, 10.7554/eLife.28533
5. Fontana, P., Buch-Larsen, S. C., Suyari, O., Smith, R., Suskiewicz, M. J., Schutzenhofer, K. *et al.* (2023) Serine ADP-ribosylation in Drosophila provides insights into the evolution of reversible ADP-ribosylation signalling Nat Commun **14**, 3200 10.1038/s41467-023-38793-y
6. Gibbs-Seymour, I., Fontana, P., Rack, J. G. M., and Ahel, I. (2016) HPF1/C4orf27 Is a PARP-1-Interacting Protein that Regulates PARP-1 ADP-Ribosylation Activity Mol Cell **62**, 432-442 10.1016/j.molcel.2016.03.008
7. Lambrecht, M. J., Brichacek, M., Barkauskaite, E., Ariza, A., Ahel, I., and Hergenrother, P. J. (2015) Synthesis of dimeric ADP-ribose and its structure with human poly(ADP-ribose) glycohydrolase J Am Chem Soc **137**, 3558-3564 10.1021/ja512528p
8. Langelier, M. F., Planck, J. L., Servent, K. M., and Pascal, J. M. (2011) Purification of human PARP-1 and PARP-1 domains from Escherichia coli for structural and biochemical analysis Methods Mol Biol **780**, 209-226 10.1007/978-1-61779-270-0_13
9. Suskiewicz, M. J., Zobel, F., Ogden, T. E. H., Fontana, P., Ariza, A., Yang, J. C. *et al.* (2020) HPF1 completes the PARP active site for DNA damage-induced ADP-ribosylation Nature **579**, 598-602 10.1038/s41586-020-2013-6
10. Rack, J. G. M., Ariza, A., Drown, B. S., Henfrey, C., Bartlett, E., Shirai, T. *et al.* (2018) (ADP-ribosyl)hydrolases: Structural Basis for Differential Substrate Recognition and Inhibition Cell Chem Biol **25**, 1533-1546 e1512 10.1016/j.chembiol.2018.11.001
11. Bonfiglio, J. J., Leidecker, O., Dauben, H., Longarini, E. J., Colby, T., San Segundo-Acosta, P. *et al.* (2020) An HPF1/PARP1-Based Chemical Biology Strategy for Exploring ADP-Ribosylation Cell **183**, 1086-1102 e1023 10.1016/j.cell.2020.09.055
12. Bartlett, E., Bonfiglio, J. J., Prokhorova, E., Colby, T., Zobel, F., Ahel, I. *et al.* (2018) Interplay of Histone Marks with Serine ADP-Ribosylation Cell Rep **24**, 3488-3502 e3485 10.1016/j.celrep.2018.08.092

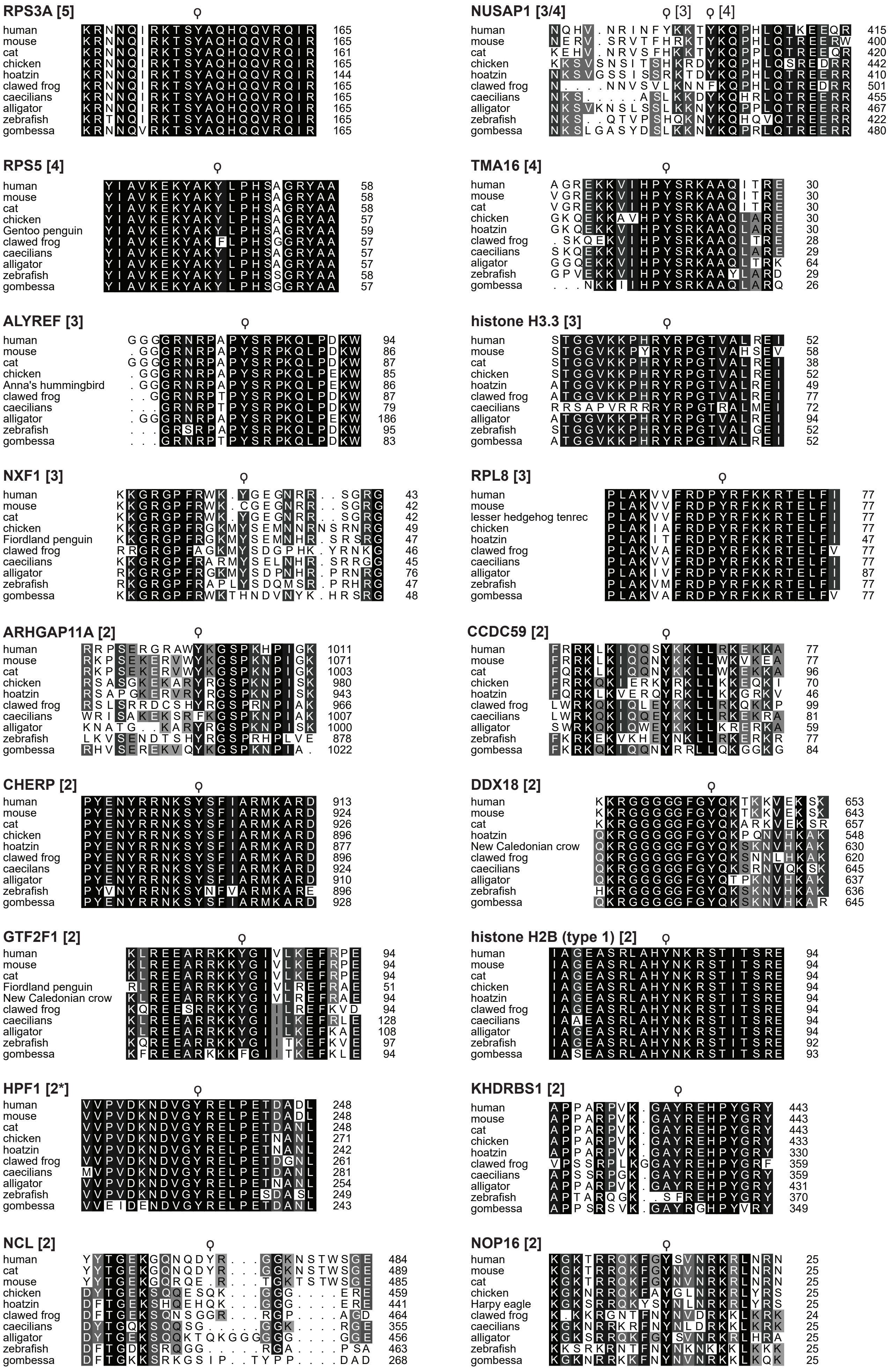
RPS3A [5]
human KRNNQIRKTSYAQHQQVRQIR 165
mouse KRNNQIRKTSYAQHQQVRQIR 165
cat KRNNQIRKTSYAQHQQVRQIR 161
chicken KRNNQIRKTSYAQHQQVRQIR 165
hoatzin KRNNQIRKTSYAQHQQVRQIR 144
clawed frog KRNNQIRKTSYAQHQQVRQIR 165
caecilians KRNNQIRKTSYAQHQQVRQIR 165
alligator KRNNQIRKTSYAQHQQVRQIR 165
zebrafish KRTNQIRKTSYAQHQQVRQIR 165
gombessa KRNNQVRKTSYAQHQQVRQIR 165
NUSAP1 [3/4]
[3] [4]
human NQHV.NRINFYKKTYKQPHLQTKEEQR 415
mouse NERV.SRVTFHRKTYKQPHLQTREERW 400
cat KEHV.NRVSFHKKTYKQPPLQTREEQR 420
chicken KKSVSNSITSHKRDYKQPHLQSREDRR 442
hoatzin NKSVGSSISSRKTDYKQPHLQTREERR 410
clawed frog N....NNVSVLKNNFKQPHLQTREDRR 501
caecilians NKS.....ASLKKDYKQHRLQTREERR 455
alligator NKSVKNSLSSLKKNYKQPPLQTREERR 467
zebrafish NKS..QTVPSHQKNYKQHQVQTREERR 422
gombessa NKSLGASYDSLKKNYKQPRLQTREERR 480
RPS5 [4]
human YIAVKEKYAKYLPHSAGRYAA 58
mouse YIAVKEKYAKYLPHSAGRYAA 58
cat YIAVKEKYAKYLPHSAGRYAA 58
chicken YIAVKEKYAKYLPHSAGRYAA 57
Gentoo penguin YIAVKEKYAKYLPHSAGRYAA 59
clawed frog YIAVKEKYAKFLPHSGGRYAA 57
caecilians YIAVKEKYAKYLPHSAGRYAA 57
alligator YIAVKEKYAKYLPHSAGRYAA 57
zebrafish YIAVKEKYAKYLPHSSGRYAA 58
gombessa YIAVKEKYAKYLPHSGGRYAA 57
TMA16 [4]
human AGREKKVIHPYSRKAAQITRE 30
mouse VGREKKVIHPYSRKAAQITRE 30
cat VGREKKVIHPYSRKAAQITRE 30
chicken GKQEKKAVHPYSRKAAQLARE 30
hoatzin GKQEKKVIHPYSRKAAQLARE 30
clawed frog .SKQEKVIHPYSRKAAQLTRE 28
caecilians SKQEKKVIHPYSRKAAQLARE 29
alligator GGQEKKVIHPYSRKAAQLTRK 64
zebrafish GPVEKKVIHPYSRKAAYLARD 29
gombessa ...NKKIIHPYSRKAAQLARQ 26
ALYREF [3]
human GGGGRNRPAPYSRPKQLPDKW 94
mouse .GGGRNRPAPYSRPKQLPDKW 86
cat GGGGRNRPAPYSRPKQLPDKW 87
chicken .GGGRNRPAPYSRPKQLPEKW 85
Anna's hummingbird .GGGRNRPAPYSRPKQLPEKW 86
clawed frog ..GGRNRPTPYSRPKQLPDKW 87
caecilians ...GRNRPTPYSRPKQLPDKW 79
alligator .GGGRNRPAPYSRPKQLPEKW 186
zebrafish ...GRSRPAPYSRPKQLPDKW 95
gombessa ...GRNRPTPYSRPKQLPDKW 83
histone H3.3 [3]
human STGGVKKPHRYRPGTVALREI 52
mouse STGGVKKPYRYRPGTVAHSEV 58
cat STGGVKKPHRYRPGTVALREI 38
chicken STGGVKKPHRYRPGTVALREI 52
hoatzin ATGGVKKPHRYRPGTVALREI 49
clawed frog ATGGVKKPHRYRPGTVALREI 77
caecilians RRSAPVRRRRYRPGTRALMEI 72
alligator ATGGVKKPHRYRPGTVALREI 94
zebrafish STGGVKKPHRYRPGTVALGEI 52
gombessa ATGGVKKPHRYRPGTVALREI 52
NXF1 [3]
human KKGRGPFRWK.YGEGNRR..SGRG 43
mouse KKGRGPFRWK.CGEGNRR..SGRG 42
cat KKGRGPFRWK.YGEGNRR..SGRG 42
chicken KKGRGPFRGKMYSEMNNRNSRNRG 49
Fiordland penguin KKGRGPFRGKMYSEMNHR.SRSRG 47
clawed frog RRGRGPFAGKMYSDGPHK.YRNKG 46
caecilians KKGRGPFRARMYSELNHR.SRRGG 45
alligator RKGRGPFRGKMYSDPNHR.PRNRG 76
zebrafish RKGRGPFRAPLYSDQMSR.PRHRG 47
gombessa KKGRGPFRWKTHNDVNYK.HRSRG 48
RPL8 [3]
human PLAKVVFRDPYRFKKRTELFI 77
mouse PLAKVVFRDPYRFKKRTELFI 77
lesser hedgehog tenrec PLAKVVFRDPYRFKKRTELFI 77
chicken PLAKIAFRDPYRFKKRTELFI 77
hoatzin PLAKITFRDPYRFKKRTELFI 47
clawed frog PLAKVAFRDPYRFKKRTELFV 77
caecilians PLAKVVFRDPYRFKKRTELFI 77
alligator PLAKIVFRDPYRFKKRTELFI 87
zebrafish PLAKVMFRDPYRFKKRTELFI 77
gombessa PLAKVAFRDPYRFKKRTELFV 77
ARHGAP11A [2]
human RRPSERGRAWYKGSPKHPIGK 1011
mouse RKPSEKERVWYKGSPKNPIGK 1071
cat RKPSEKERVWYKGSPKNPIGK 1003
chicken RSASGKEKARYRGSPKNPISK 980
hoatzin RSAPGKERVRYRGSPKNPISK 943
clawed frog RSLSRRDCSHYRGSPRNPIAK 966
caecilians WRISAKEKSRFKGSPKNPIAK 1007
alligator KNATG..KARYRGSPKNPISK 1000
zebrafish LKVSENDTSHYRGSPRHPLVE 878
gombessa RHVSEREKVQYKGSPKNPIA. 1022
CCDC59 [2]
human FRRKLKIQQSYKKLLRKEKKA 77
mouse FRRKLKIQQNYKKLLWKVKEA 77
cat FQRKLKIQQNYKKLLWKEKKA 96
chicken FRRKQKIERKYRKLLKKEQKI 70
hoatzin FQRKLKVERQYRKLLKKGRKV 46
clawed frog LWRKQKIQLEYKKLLRKQKKP 99
caecilians LWRKQKIQQEYKKLLRKEKRA 81
alligator SWRKQKIQWEYKKLLKRERKA 59
zebrafish FKRKEKVKHEYNKLLRKERKR 77
gombessa FKRKQKIQQNYRRLLQKGGKG 84
CHERP [2]
human PYENYRRNKSYSFIARMKARD 913
mouse PYENYRRNKSYSFIARMKARD 924
cat PYENYRRNKSYSFIARMKARD 926
chicken PYENYRRNKSYSFIARMKARD 896
hoatzin PYENYRRNKSYSFIARMKARD 877
clawed frog PYENYRRNKSYSFIARMKARD 896
caecilans PYENYRRNKSYSFIARMKARD 924
alligator PYENYRRNKSYSFIARMKARD 910
zebrafish PYVNYRRNKSYNFVARMKARE 896
gombessa PYENYRRNKSYSFIARMKARD 928
DDX18 [2]
human KKRGGGGGFGYQKTKKVEKSK 653
mouse KKRGGGGGFGYQKTKKVEKSK 643
cat KKRGGGGGFGYQKARKVEKSR 657
hoatzin QKRGGGGGFGYQKPQNVHKAK 548
New Caledonian crow QKRGGGGGFGYQKSKNVHKAK 630
clawed frog QKRGGGGGFGYQKSNNLHKAK 620
caecilians QKRGGGGGFGYQKSRNVQKSK 645
alligator QKRGGGGGFGYQTPKNVHKAK 637
zebrafish HKRGGGGGFGYQKSKNVHKAK 636
gombessa QKRGGGGGFGYQKSKNVHKAR 645
GTF2F1 [2]
human KLREEARRKKYGIVLKEFRPE 94
mouse KLREEARRKKYGIVLKEFRPE 94
cat KLREEARRKKYGIVLKEFRPE 94
Fiordland penguin RLREEARRKKYGIVLREFRAE 51
New Caledonian crow KLREEARRKKYGIVLREFRAE 94
clawed frog KQREESRRKKYGIILREFKVD 94
caecilians KLREEARRKKYGIILKEFRLE 128
alligator KLREEARRKKYGIILKEFKAE 108
zebrafish KQREEARRKKYGIITKEFKVE 97
gombessa KFREEARKKKFGIITKEFKLE 94
histone H2B (type 1) [2]
human IAGEASRLAHYNKRSTITSRE 94
mouse IAGEASRLAHYNKRSTITSRE 94
cat IAGEASRLAHYNKRSTITSRE 94
chicken IAGEASRLAHYNKRSTITSRE 94
hoatzin IAGEASRLAHYNKRSTITSRE 94
clawed frog IAGEASRLAHYNKRSTITSRE 94
caecilians IAAEASRLAHYNKRSTITSRE 94
alligator IAGEASRLAHYNKRSTITSRE 94
zebrafish IAGEASRLAHYNKRSTITSRE 92
gombessa IASEASRLAHYNKRSTITSRE 93
HPF1 [2*]
human VVPVDKNDVGYRELPETDADL 248
mouse VVPVDKNDVGYRELPETDADL 248
cat VVPVDKNDVGYRELPETDANL 248
chicken VVPVDKNDVGYRELPETNANL 271
hoatzin VVPVDKNDVGYRELPETNANL 242
clawed frog VVPVDKNDVGYRELPETDGNL 261
caecilians MVPVDKNDVGYRELPETDANL 281
alligator VVPVDKNDVGYRELPETNANL 254
zebrafish VVPVDKNDVGYRELPESDASL 249
gombessa VVEIDENDVGYRELPETDANL 243
KHDRBS1 [2]
human APPARPVK.GAYREHPYGRY 443
mouse APPARPVK.GAYREHPYGRY 443
cat APPARPVK.GAYREHPYGRY 443
chicken APPARPVK.GAYREHPYGRY 433
hoatzin APPARPVK.GAYREHPYGRY 330
clawed frog VPSSRPLKGGAYREHPYGRF 359
caecilians APSSRPGK.GAYREHPYGRY 359
alligator APPARPVK.GAYREHPYGRY 431
zebrafish APTARQGK..SFREHPYGRY 370
gombessa APPSRSVK.GAYRGHPYVRY 349
NCL [2]
human YYTGEKGQNQDYR...GGKNSTWSGE 484
cat YYTGEKGQNQDYR...GGKNSTWSGE 489
mouse YYTGEKGQRQE.R...TGKTSTWSGE 485
chicken DYTGEKSQQESQK...GGG....ERE 459
hoatzin DFTGEKSHQEHQK...GGG....ERE 441
clawed frog DFTGEKSQNSGGR...RGP....AGD 464
caecilians DYTGQKSQQSG.....GGK....RGE 355
alligator DYTGEKSQQKTQKGGGGGG....GGE 456
zebrafish DFTGDKSRQGG.....RGA....PSA 463
gombessa DFTGKKSRKGSIP..TYPP....DAD 268
NOP16 [2]
human KGKTRRQKFGYSVNRKRLNRN 25
mouse KGKTRRQKFGYNVNRKRLNRN 25
cat KGKTRRQKFGYNVNRKRLNRN 25
chicken KGKNRRQKFAYGLNRKRLYRS 25
Harpy eagle KGKSRRQKYSYNLNRKRLYRS 25
clawed frog K.KKRGNTFNYNVDRKKLKRK 24
caecilians KGKNRRKRFNYNLDRKKLKRK 25
alligator KGKSRRQKFGYSVNRKRLHRA 25
zebrafish KKSRKRNTFNYNKNKKKLKKK 25
gombessa KGKNRRKKFNYNVNRKKLKQK 25

Figure S1 (1/2)

PPHLN1 [2]

human	MRDGFRRKSFYSSH.YARERSPYKRDNTFFRES	133
mouse	MRDGFRRKSFYSSH.YSRDRSPHKRDAPFFRES	147
cat	MRDGFRRKSFYPSH.YARDRSPHKRDPPFFRES	140
chicken	VRDGFRRKSFHPSH.YMRERSPHKRDSPFFRES	145
hoatzin	VRDGFRRKSFYPSH.YMRERSPHKRDSPFFRES	135
clawed frog	SKDAYKKKPYYHPNICPRERSPHRRDSPFVRDS	136
caecilians	SREGFRRKPFYPPH..IRDRSPQKRDSPYFRES	160
alligator	IRDGFRRKSFYPSH.YIRERSPHKRDSPFFRES	142
zebrafish	ERENVRRKGPFPP...VRERSPARRDIP.....	184
gombessa	SRETFRRKGFNLHP.HARDRSPHKRGPLLYRGS	148

RFC1 [2]

human	SEKKRTNYQAYRSYLNREGPK	392
mouse	SEKKRTNYQAYRSYLNREGPK	389
cat	SEKRRTNYQAYRSYLNREGPK	387
chicken	SEKRRNNYQAYRSFLNREGPK	400
hoatzin	SEKKRINYQAYRSFLNREGPK	420
clawed frog	SEKKRANFKAYQNFLNREGPK	384
caecilians	SEKKRVNFQAYRNFLNREGPK	423
alligator	SEKKRVNYQAYRSFLNREGPK	387
zebrafish	LEKKRANSSAYRSYLNREGPR	388
gombessa	SEKKRVNYQAYRNFLNREGPK	393

RPL29[¶] [2]

human	GVSRKLDRLAYIAHPKLGKRA	108
mouse	G..PKLKRLAFIAHPKLGKRI	106
cat	GGSRKLNRLAYIAHPKLGKRA	108
cattle	GGSRKLSRLAYIAHPKLGKRA	108
narwal	GGSRKLSRLAYIAHPRLGKRA	108
West Indian manatee	GVNRKLSRLAYIAHPKLGKRA	108
lesser hedgehog tenrec	GFNRNLSCLAYIAHPKLGKRA	112

RPL35 [2]

human	TKKQQRKERLYPLRKYAVKA	123
mouse	TKKQQRKERLYPLRKYAVKA	123
cat	TKKQQRKERLYPLRKYAVKA	123
chicken	TKKQQRKERLYPVRKYAIKA	123
hoatzin	TKKQQRKERLYPMRKYAIKA	98
clawed frog	TKKQQRKDRLYSVRKFAVKA	123
caecilians	TKKQQRKERLYPMRKFAVKA	123
alligator	TKKQQRKERLYPVRKYAVKA	123
zebrafish	TKKMQRKSRLYSIRKFAVKA	123
gombessa	TKKQQRKERLYPMRKFAVKA	123

SF3B2 [2]

human	PQDSRGGSKKYKEFKF	895
mouse	PQDSRGGSKKYKEFKF	878
cat	PQDSRGGSKKYKEFKF	896
white-rumped snowfinch	PQDARGGGKKYKEFKF	749
Fiordland penguin	PQDARGGGKKYKEFKF	731
alligator	PQDARGGGKKYKEFKF	895
clawed frog	PQDSRAGGKKYKEFKF	764
caecilians	PQDTRGGGKKYKEFKF	874
zebrafish	PQDTRGGAKKYKEFKF	825
gombessa	PQDTKGGAKKYKEFKF	856

ZnF182[π] [2]

human	ECEKAFSQKSYLMLHQRGHTG	482
cat	ECEKAFSQKSYLMLHQRGHTG	465
mouse	ECEKAFSQKSYLMLHQRGHTG	538
narwal	ECEKAFSQKSYLMLHQRGHTG	463
lesser hedgehog tenrec	ECEKAFSQKSYLMLHQRGHTG	465
Linnaeus's two-toed sloth	ECEKAFSQKSYLMLHQRGHTG	489

Figure S1 (2/2)

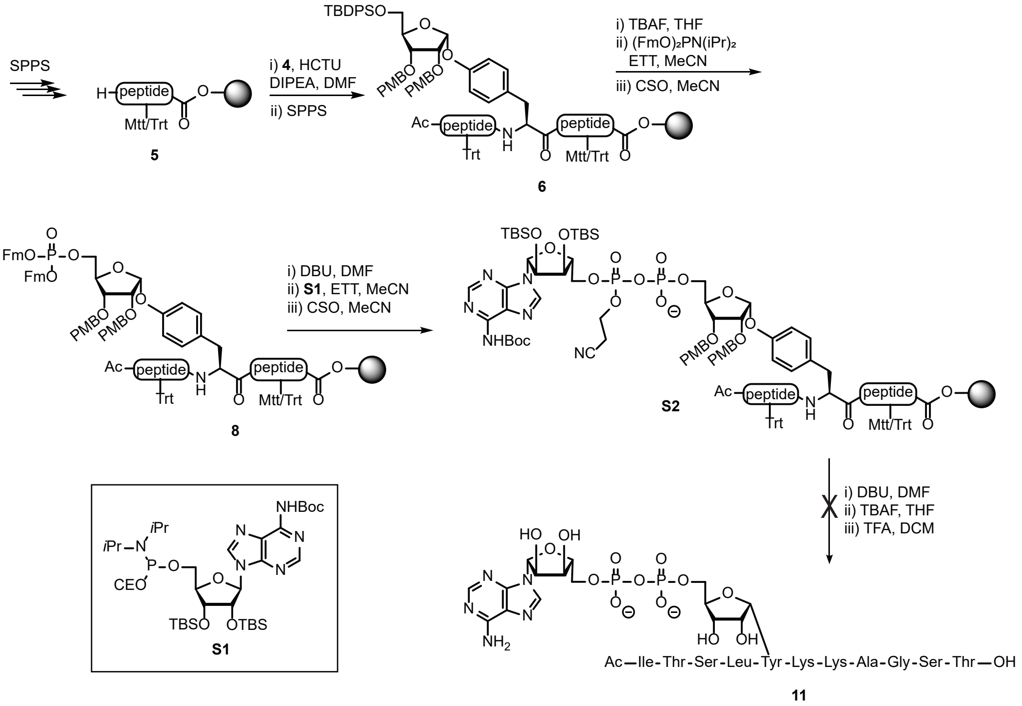
SPPS
H-peptide
Mtt/Trt
5
i) 4, HCTU
DIPEA, DMF
ii) SPPS
TBDPSO
PMBO
Ac-peptide
Trt
6
i) TBAF, THF
ii) (FmO)2PN(iPr)2
ETT, MeCN
iii) CSO, MeCN
FmO
8
i) DBU, DMF
ii) S1, ETT, MeCN
iii) CSO, MeCN
TBSO
OTBS
NHBoc
NC
S2
i) DBU, DMF
ii) TBAF, THF
iii) TFA, DCM
iPr
CEO
TBSO
S1
HO
OH
NH2
Ac–Ile-Thr-Ser-Leu-Tyr-Lys-Lys-Ala-Gly-Ser-Thr–OH
11

Figure S2

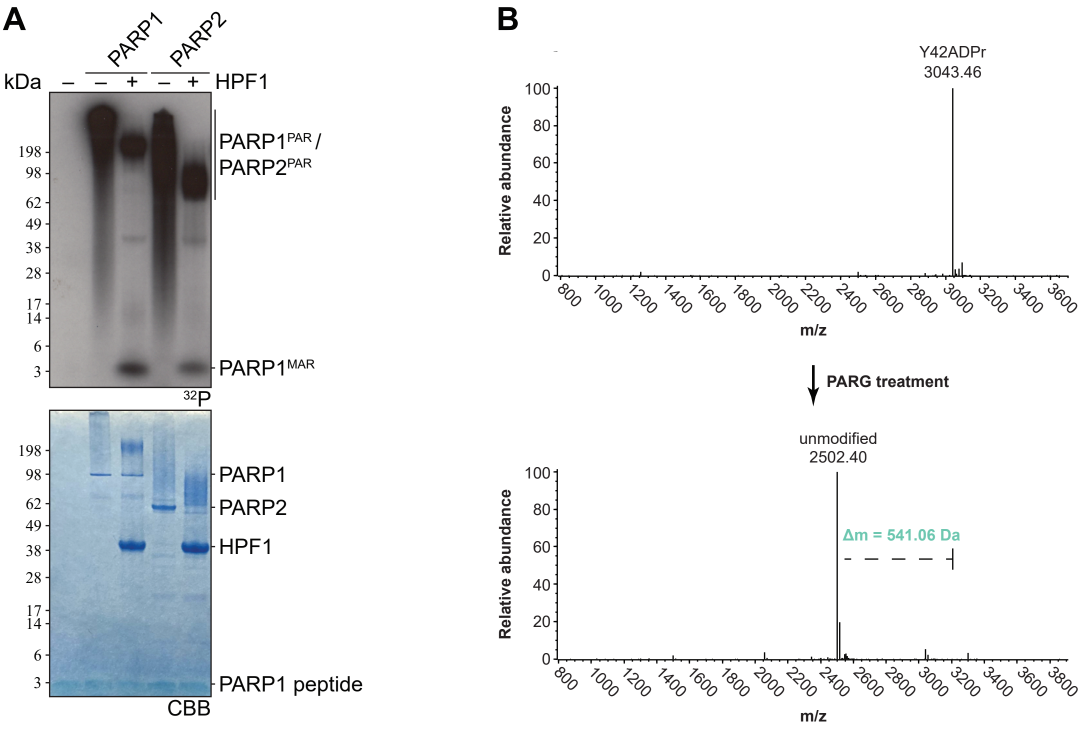
A
PARP1
PARP2
kDa
HPF1
PARP1PAR /
PARP2PAR
PARP1MAR
32P
PARP1
PARP2
HPF1
PARP1 peptide
CBB
B
Y42ADPr
3043.46
Relative abundance
m/z
PARG treatment
unmodified
2502.40
Δm = 541.06 Da
Relative abundance
m/z

Figure S3

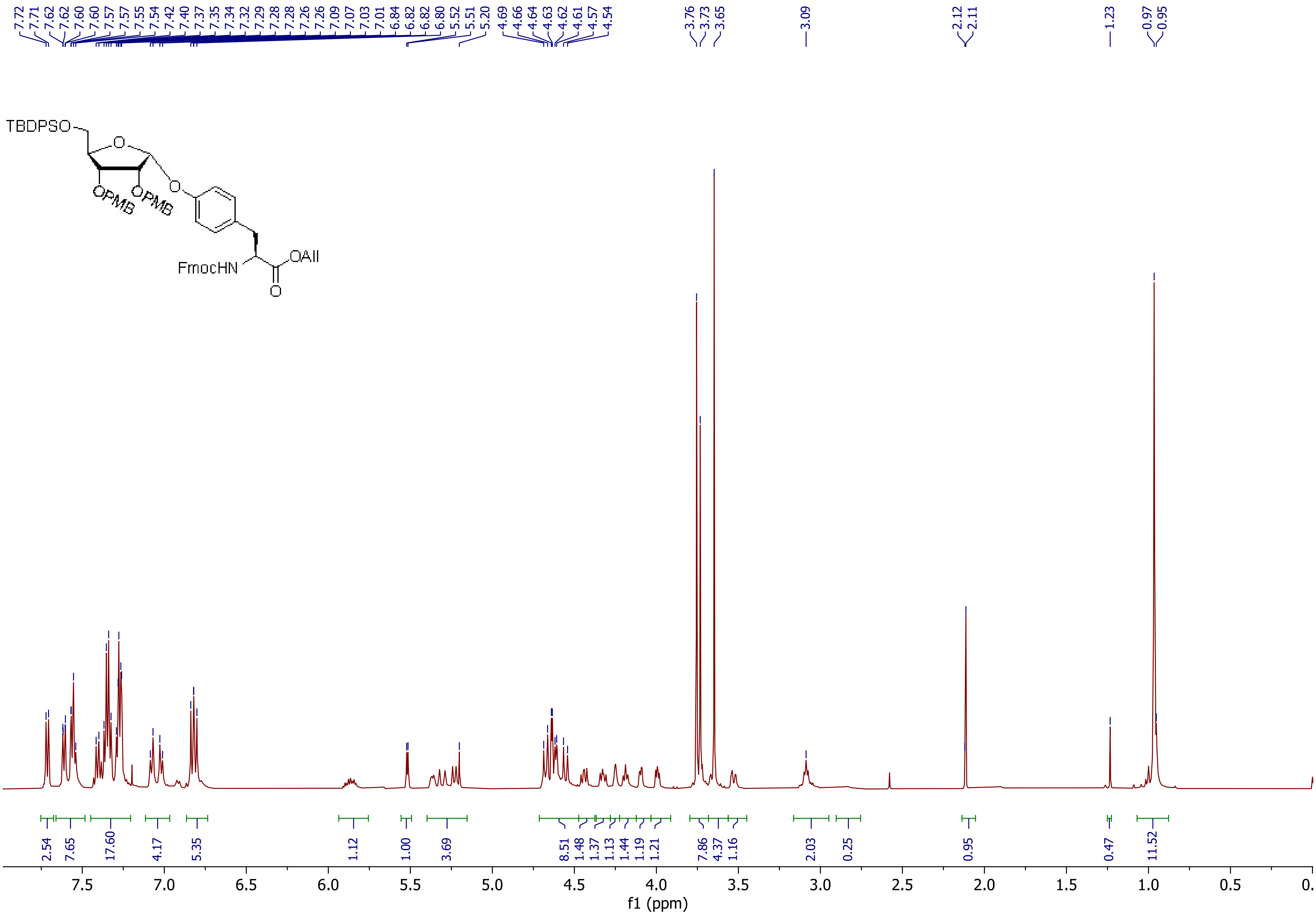

TBDPSO
O
OPMB
OPMB
O
FmocHN
OAll
O
7.72 7.71 7.62 7.62 7.60 7.57 7.57 7.55 7.54 7.42 7.40 7.37 7.35 7.34 7.32 7.29 7.28 7.28 7.26 7.26 7.09 7.07 7.03 7.01 6.84 6.82 6.82 6.80 5.52 5.51 5.20 4.69 4.66 4.64 4.63 4.62 4.61 4.57 4.54
3.76 3.73 3.65
3.09
2.12 2.11
1.23
0.97 0.95
2.54 7.65 17.60 4.17 5.35 1.12 1.00 3.69 8.51 1.48 1.37 1.13 1.44 1.19 1.21 7.86 4.37 1.16 2.03 0.25 0.95 0.47 11.52
7.5 7.0 6.5 6.0 5.5 5.0 4.5 4.0 3.5 3.0 2.5 2.0 1.5 1.0 0.5 0.
f1 (ppm)

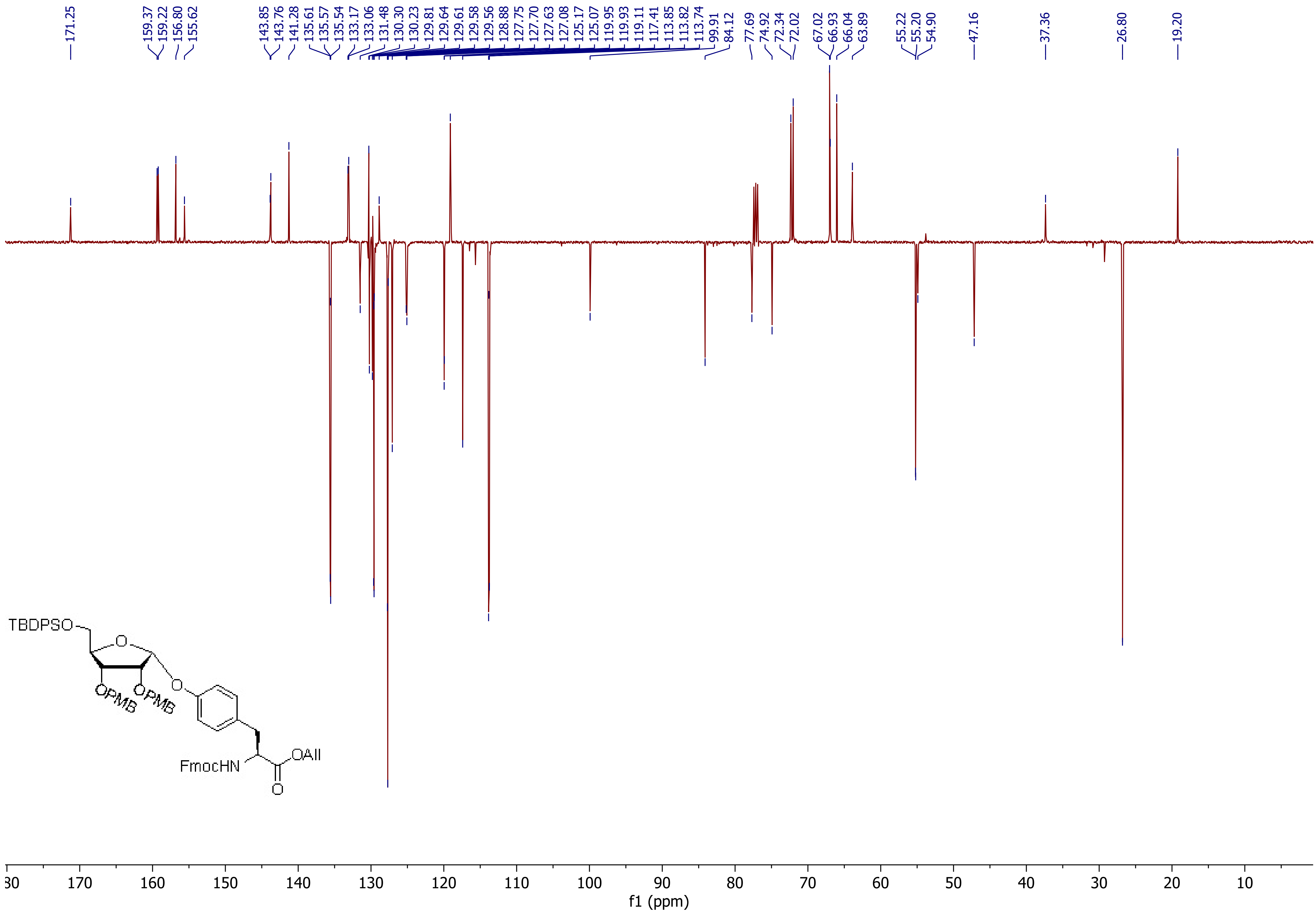

171.25
159.37
159.22
156.80
155.62
143.85
143.76
141.28
135.61
135.57
135.54
133.17
133.06
131.48
130.30
130.23
129.81
129.64
129.61
129.58
129.56
128.88
127.75
127.70
127.63
127.08
125.17
125.07
119.95
119.93
119.11
117.41
113.85
113.82
113.74
99.91
84.12
77.69
74.92
72.34
72.02
67.02
66.93
66.04
63.89
55.22
55.20
54.90
47.16
37.36
26.80
19.20
TBDPSO
O
OPMB
OPMB
O
FmocHN
OAll
O
30
170
160
150
140
130
120
110
100
90
80
70
60
50
40
30
20
10
f1 (ppm)

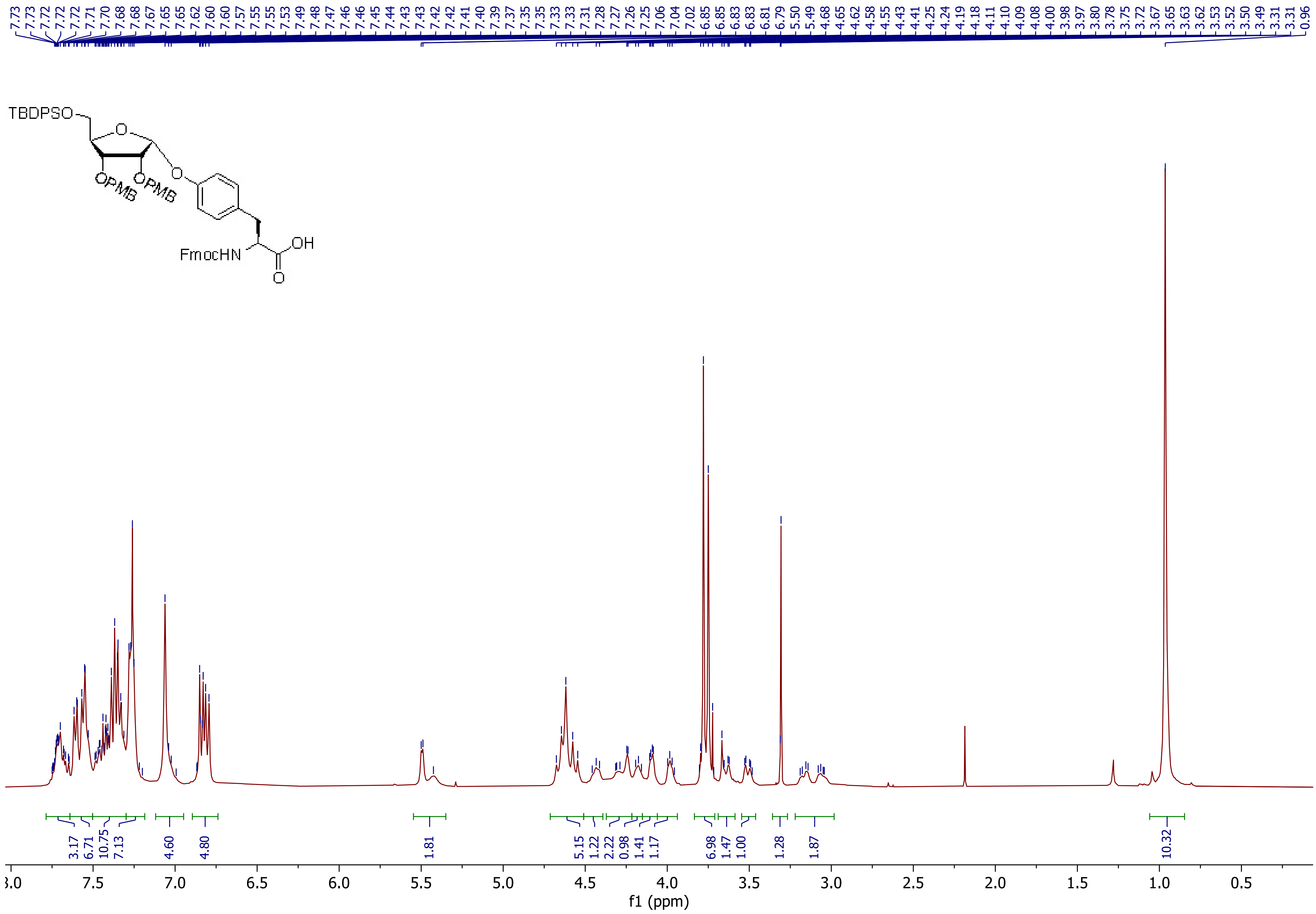
7.73 7.73 7.72 7.72 7.72 7.71 7.70 7.68 7.68 7.67 7.65 7.65 7.62 7.60 7.60 7.57 7.55 7.55 7.53 7.49 7.48 7.47 7.46 7.46 7.45 7.44 7.43 7.43 7.42 7.42 7.41 7.40 7.39 7.37 7.35 7.35 7.33 7.33 7.31 7.28 7.27 7.26 7.25 7.06 7.04 7.02 6.85 6.85 6.83 6.83 6.81 6.79 5.50 5.49 4.68 4.65 4.62 4.58 4.55 4.43 4.41 4.25 4.24 4.19 4.18 4.11 4.10 4.09 4.08 4.00 3.98 3.97 3.80 3.78 3.75 3.72 3.67 3.65 3.63 3.62 3.53 3.52 3.50 3.49 3.31 3.31 0.96
TBDPSO
O
O
OPMB
OPMB
FmocHN
OH
O
3.17
6.71
10.75
7.13
4.60
4.80
1.81
5.15
1.22
2.22
0.98
1.41
1.17
6.98
1.47
1.00
1.28
1.87
10.32
7.5 7.0 6.5 6.0 5.5 5.0 4.5 4.0 3.5 3.0 2.5 2.0 1.5 1.0 0.5
f1 (ppm)

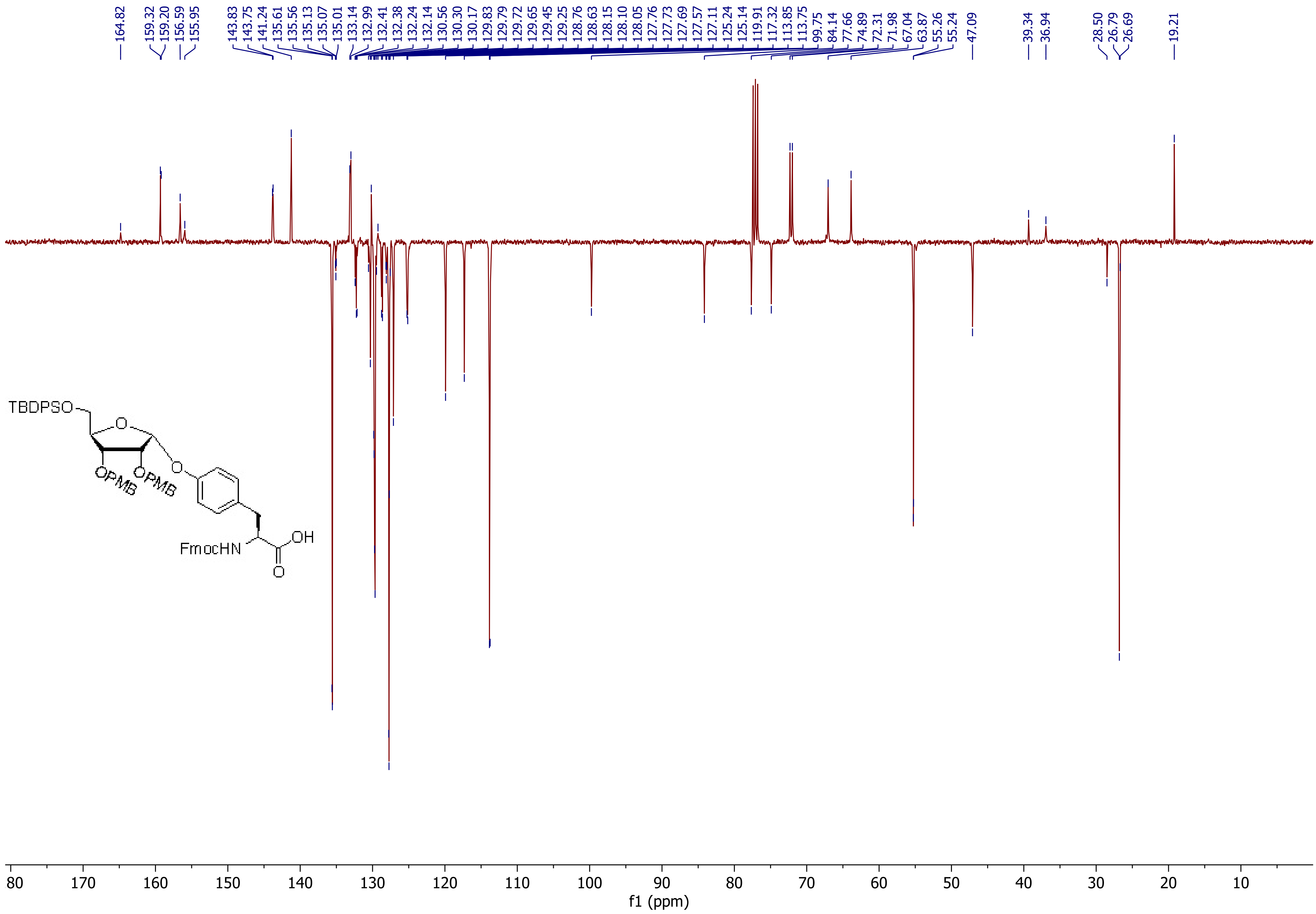

164.82
159.32
159.20
156.59
155.95
143.83
143.75
141.24
135.61
135.56
135.13
135.07
135.01
133.14
132.99
132.41
132.38
132.24
132.14
130.56
130.30
130.17
129.83
129.79
129.72
129.65
129.45
129.25
128.76
128.63
128.15
128.10
128.05
127.76
127.73
127.69
127.57
127.11
125.24
125.14
119.91
117.32
113.85
113.75
99.75
84.14
77.66
74.89
72.31
71.98
67.04
63.87
55.26
55.24
47.09
39.34
36.94
28.50
26.79
26.69
19.21
TBDPSO
O
OPMB
OPMB
O
FmocHN
OH
O
80
170
160
150
140
130
120
110
100
90
80
70
60
50
40
30
20
10
f1 (ppm)

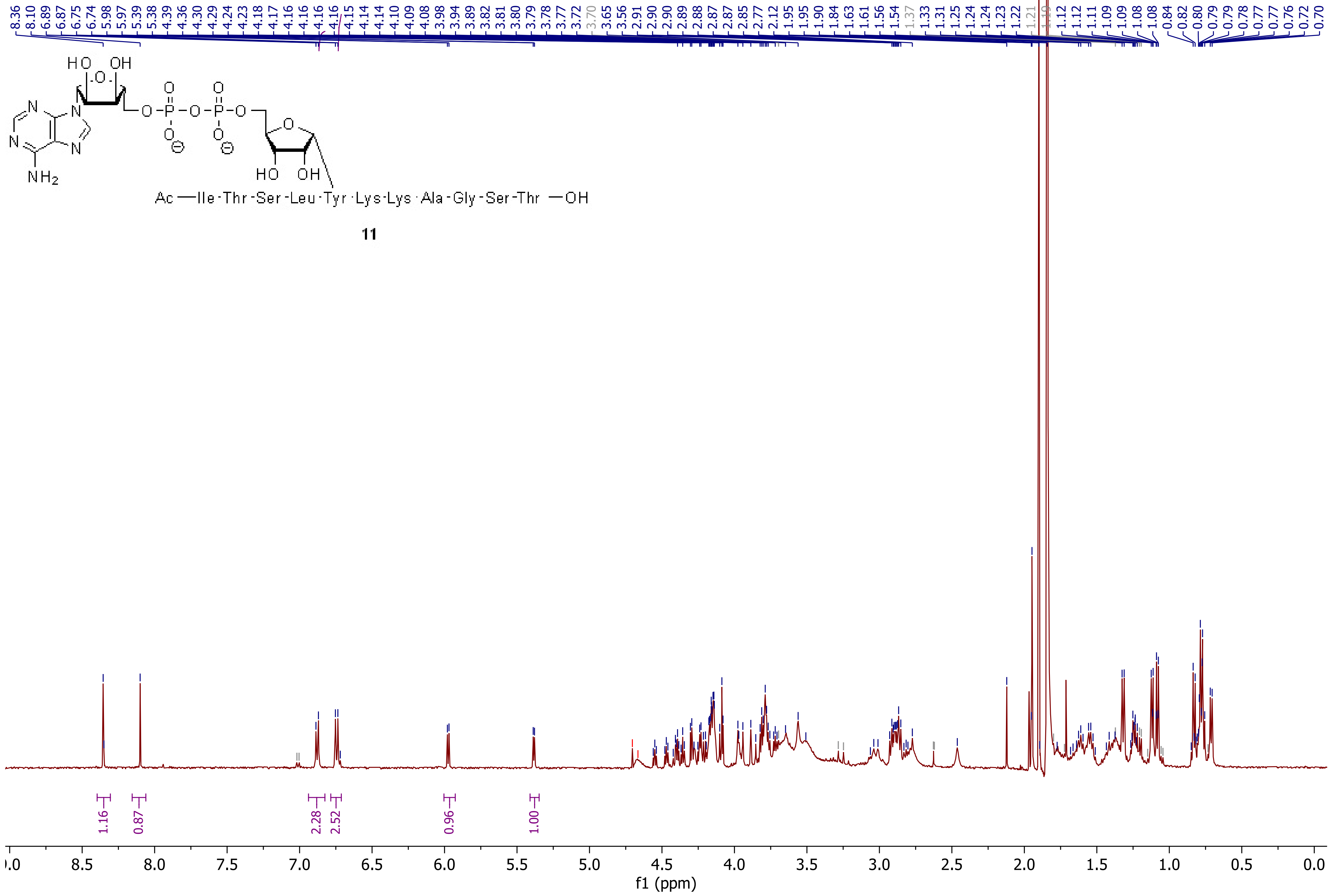
8.36 8.10 6.89 6.87 6.75 6.74 5.98 5.97 5.39 5.38 4.39 4.36 4.30 4.29 4.24 4.23 4.18 4.17 4.16 4.16 4.16 4.16 4.15 4.14 4.14 4.10 4.09 4.08 3.98 3.94 3.89 3.82 3.81 3.80 3.79 3.78 3.77 3.72 3.70 3.65 3.56 2.91 2.90 2.90 2.89 2.88 2.87 2.87 2.85 2.77 2.12 1.95 1.95 1.90 1.84 1.63 1.61 1.56 1.54 1.37 1.33 1.31 1.25 1.24 1.24 1.23 1.22 1.21 1.19 1.12 1.12 1.11 1.09 1.09 1.08 1.08 0.84 0.82 0.80 0.79 0.79 0.78 0.77 0.77 0.76 0.72 0.70
HO OH
O
N
N
N
N
N
NH2
O-P-O-P-O
O O
HO OH
Ac —Ile-Thr-Ser-Leu-Tyr-Lys-Lys-Ala-Gly-Ser-Thr —OH
11
1.16 0.87 2.28 2.52 0.96 1.00
9.0 8.5 8.0 7.5 7.0 6.5 6.0 5.5 5.0 4.5 4.0 3.5 3.0 2.5 2.0 1.5 1.0 0.5 0.0
f1 (ppm)

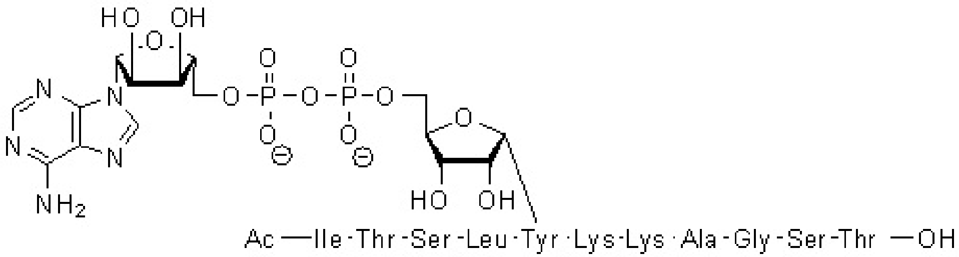
HO OH
O
N
N
N
N
N
NH2
O-P-O-P-O
O
O
HO OH
Ac —Ile·Thr·Ser·Leu·Tyr·Lys·Lys·Ala·Gly·Ser·Thr —OH

11

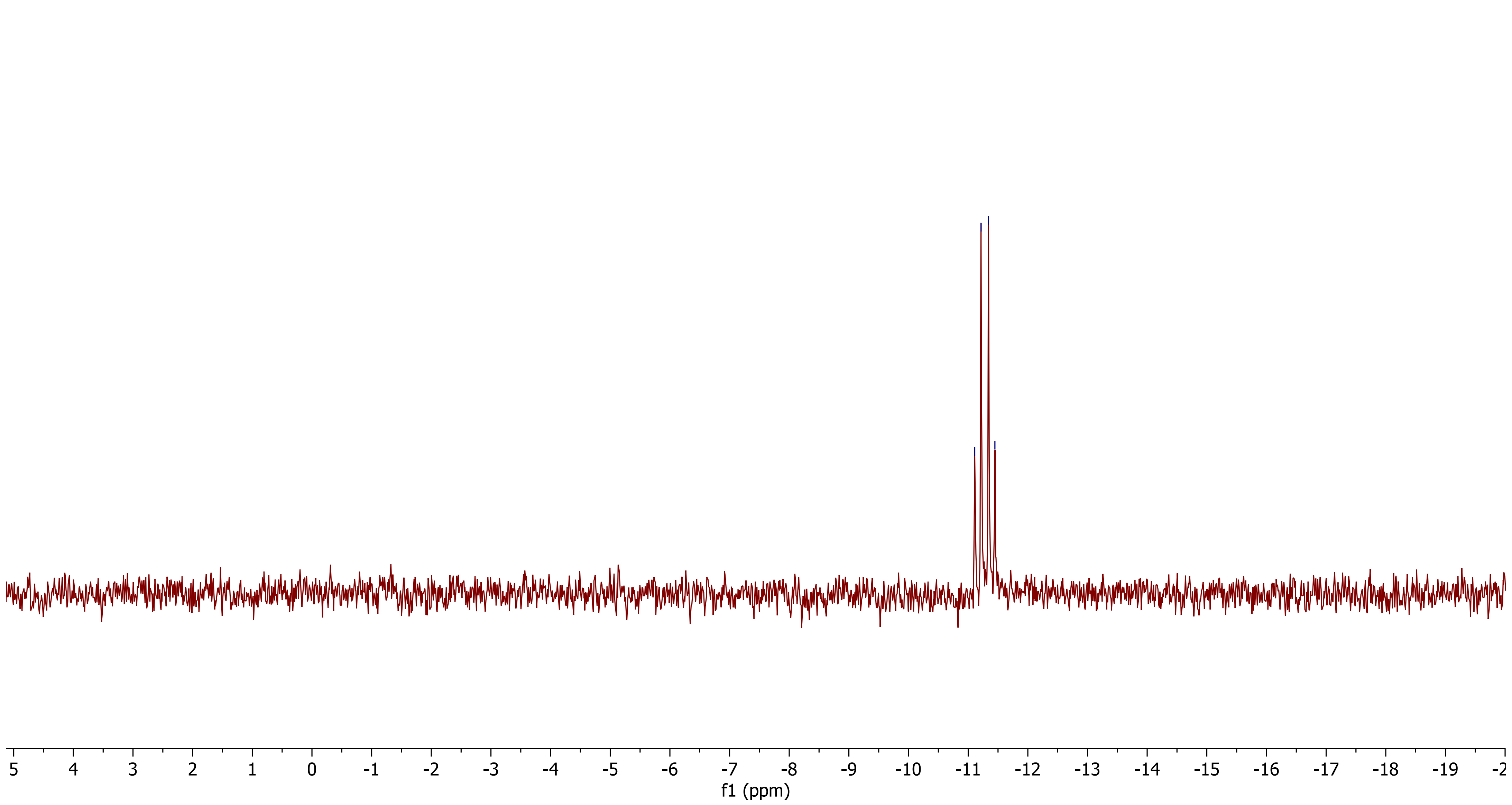
-11.11
-11.22
-11.34
-11.45
5 4 3 2 1 0 -1 -2 -3 -4 -5 -6 -7 -8 -9 -10 -11 -12 -13 -14 -15 -16 -17 -18 -19
f1 (ppm)
